# Tracking the mechanism of covalent molecular glue stabilization using native mass spectrometry†

**DOI:** 10.1039/d3sc01732j

**Published:** 2023-05-31

**Authors:** Carlo J. A. Verhoef, Danielle F. Kay, Lars van Dijck, Richard G. Doveston, Luc Brunsveld, Aneika C. Leney, Peter J. Cossar

**Affiliations:** a Department of Biomedical Engineering and Institute for Complex Molecular Systems, Eindhoven University of Technology Eindhoven 5600 MB The Netherlands p.cossar@tue.nl; b School of Biosciences, University of Birmingham Birmingham B15 2TT UK a.leney@bham.ac.uk; c Leicester Institute of Structural and Chemical Biology and School of Chemistry, University of Leicester Leicester LE1 7RH UK

## Abstract

Molecular glues are powerful tools for the control of protein–protein interactions. Yet, the mechanisms underlying multi-component protein complex formation remain poorly understood. Native mass spectrometry (MS) detects multiple protein species simultaneously, providing an entry to elucidate these mechanisms. Here, for the first time, covalent molecular glue stabilization was kinetically investigated by combining native MS with biophysical and structural techniques. This approach elucidated the stoichiometry of a multi-component protein–ligand complex, the assembly order, and the contributions of covalent *versus* non-covalent binding events that govern molecular glue activity. Aldehyde-based molecular glue activity is initially regulated by cooperative non-covalent binding, followed by slow covalent ligation, further enhancing stabilization. This study provides a framework to investigate the mechanisms of covalent small molecule ligation and informs (covalent) molecular glue development.

## Abstract 2

The molecular mechanisms underlying molecular glue protein complex stabilization are poorly understood. In this study, we kinetically investigate covalent molecular glue stabilization of a canonical protein–protein interaction using native mass spectrometry.

## Graphical abstract

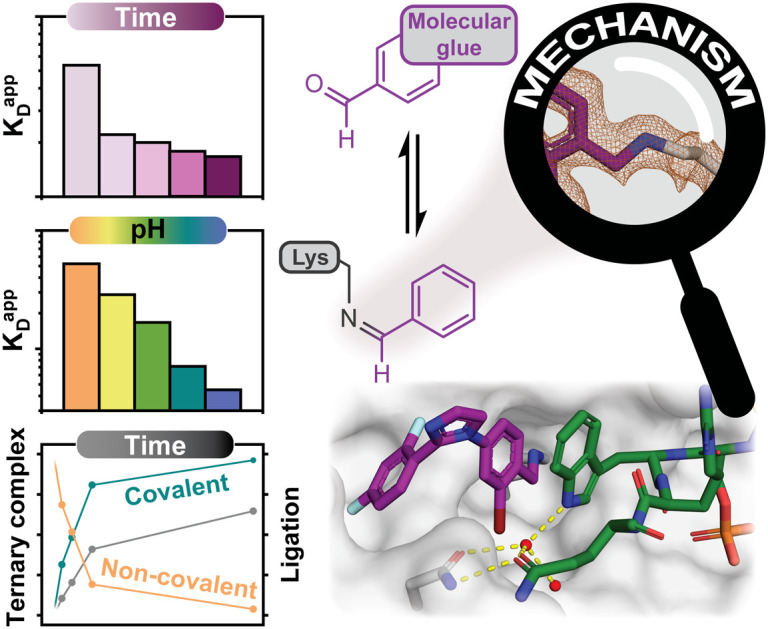

## Introduction

Molecular glues have revolutionized drug discovery by enabling greater control of protein function through chemical-induced protein complexation.^1,2^ These small molecules enhance protein complex avidity by influencing the protein–protein interaction (PPI) interface. This drug discovery approach translates to greater control of the proteome, for instance, *via* introducing new cellular functions,^3,4^ generating dead-end protein complexes,^5,6^ modulating protein localization,^7^ or restoration^8^/enhancement^9^ of protein function. This approach has enabled the targeting of proteins once considered undruggable by addressing a protein complex in contrast to a dysregulated protein in isolation.^10,11^

Recently, the fusion of covalent and molecular glue drug discovery has yielded a new and promising alternative drug discovery approach.^1,12^ Briefly, covalent molecular glues incorporate an electrophile that reacts with a nucleophilic amino acid on one of the protein partners. Covalent molecular glues EN450 (ref. 13) and RM-18 (ref. 5 and 6) stand as compelling examples of the potential of this technology. Further, reversible covalent electrophiles, including disulfides^14–16^ and imines,^17^ show promise for fragment-based molecular glue development, providing initial chemical matter for further drug optimization.

While covalent molecular glues show potential, the mechanistic understanding by which covalent molecular glues stabilize multi-component protein complexes remains limited. This stabilization process consists of sequential non-covalent and covalent binding events between the individual proteins and the molecular glue. Traditional biochemical assays report globally on PPI formation, providing limited information regarding the order of multi-component protein complex assembly, and typically do not distinguish between non-covalent and covalent binding events. Native mass spectrometry provides a solution to study these molecular processes.

Significant advances in native mass spectrometry (MS) have enabled the high-resolution detection of protein complexes in their native state, enabling protein function elucidation.^18,19^ Further, native MS is a powerful tool to investigate ligand–protein complexes,^20–22^ ligand-DNA complexes,^23^ and molecular glue protein complex stabilization.^24,25^ In contrast to protein denaturing MS techniques, native MS preserves protein complexes from solution into the gas phase enabling the analyses of protein complexes in their native state.^18,19^ This provides macromolecular insight into the abundances of individual proteins and protein complexes, their affinity, and the stoichiometry of the PPIs. Previously, native MS/MS experiments using antibody–drug conjugates have demonstrated that non-covalent interactions can be disrupted, whilst covalent interactions between the bioconjugate are maintained.^26^ This technique presents an exciting opportunity for covalent drug discovery, as covalent and non-covalent binding modes can be probed using native MS.

Here we elucidate that aldehyde-based molecular glue stabilization occurs *via* a two-step process using a time-dependent fluorescence anisotropy (td-FA) assay. We then applied native MS experiments to disentangle the stepwise covalent stabilization mechanism of a molecular glue, for the first time. Specifically, by developing a native MS/MS method, non-covalent and covalent interactions were discriminated allowing the assembly of the ternary protein complex to be tracked over time, and as a function of covalent ligation. These data provided a deep molecular understanding of covalent molecular glue stabilization, showing that aldehyde-based molecular glue induced stabilization is composed of highly cooperative non-covalent binding followed by slow covalent ligation.

## Results and discussion

### Aldehyde-MG stabilization of 14-3-3 complexes proceeds *via* a two-phase process

Covalent molecular glue drug discovery has emerged as a promising approach. However, to date studies, including our own research using disulfide,^14,27^ and aldehyde-based chemistry^17,27^ have focused on understanding structure–activity relationships with limited to no consideration of the kinetics of covalent molecular glue stabilization.

**Fig. 1:**
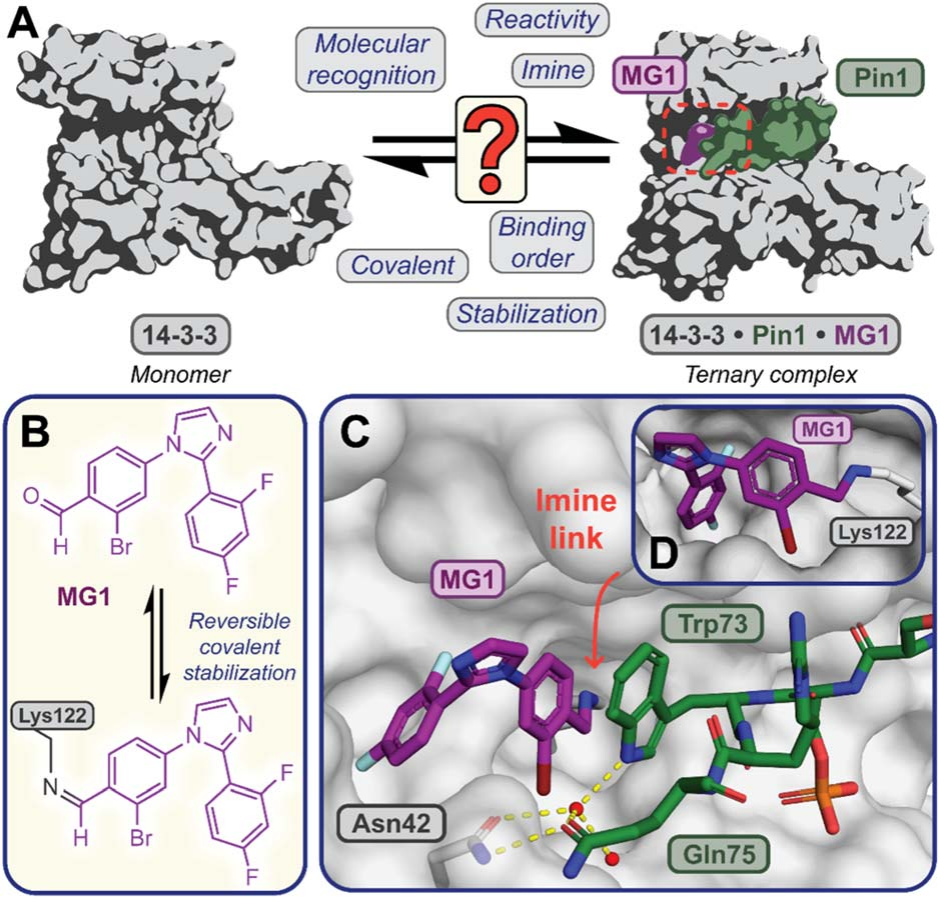
(A) Illustrates the limited understanding of MG1 induced complex stabilization. (B) Schematic representation of MG1 ligation. (C) Enlarged view of the 14-3-3/Pin1/MG1 interface (PDB : 7BFW, previous work^17^). (D) MG1 covalently bound to Lys122 of 14-3-3 by aldimine bonding.^17^

**Fig. 2:**
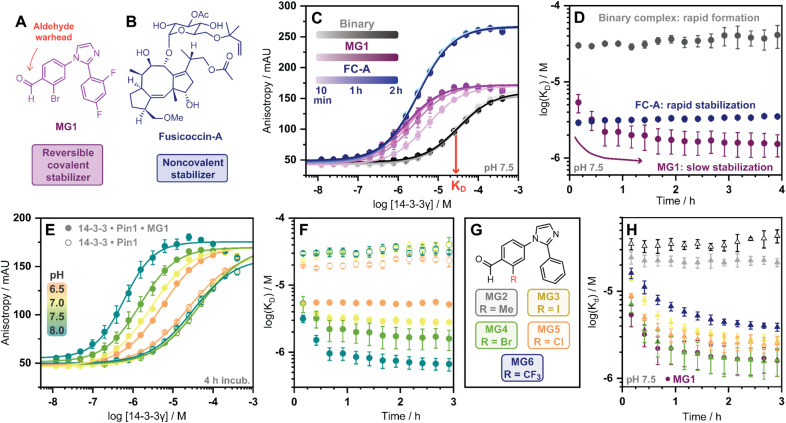
(A and B) Chemical structures of MG1 and FC-A. (C) 14-3-3γ titration FA assay, using fluorescein-Pin1 peptide in absence (grey), or presence of molecular glue MG1 (purple) or FC-A (blue) over time. (D) *K*_D_^app^ plot over time of 14-3-3/Pin1 (grey), 14-3-3/Pin1/FC-A (blue) and 14-3-3/Pin1/MG1 (purple) complexes. (E) 14-3-3γ titration over the pH range 6.5–8.0 in absence (hollow dots), or presence of molecular glue MG1 (solid dots). (F) *K*_D_^app^ plot over time of 14-3-3/Pin1 (hollow dots) and 14-3-3/Pin1/MG1 (solid dots) complexes over pH range 6.5–8.0. (G) The panel of 2-ortho substituted benzaldehydes. (H) *K*_D_^app^ plot over time of the 14-3-3/Pin1 complex (hollow triangles), MG1 stabilized 14-3-3/Pin1 complex (solid dots), and MG2–6 stabilized 14-3-3/Pin1 complexes (solid triangles). Error bars represent the standard deviation (*n* = 3).

To investigate the kinetics of molecular glue induced protein complex stabilization, and in turn the underlying molecular mechanism (Fig. 1A), we utilized aldehyde-based molecular glue MG1 as a case study (Fig. 1B).^17^ As MG1 has previously been extensively biochemically and structurally characterized, this provided a robust base for systematically understanding the kinetic processes that lead to covalent molecular glue stabilization. MG1 stabilizes the hub protein 14-3-3/peptidyl-prolyl *cis*–*trans* isomerase NIMA-interacting 1 (Pin1) PPI (Fig. 1A–D and 2A). This small molecule forms an imine bond between the formyl group of MG1 and Lys122 of 14-3-3 (Fig. S1A–C†).^17^ Notably, 14-3-3 is a dimeric protein with each monomer binding a single phospho-peptide and molecular glue, independent of the complementary monomer (Fig. S1D†). Given this independent binding behaviour of the 14-3-3 monomers, the crystal structure of 14-3-3 is depicted as a monomer.

Cognizant that small molecule imine bond formation typically takes minutes to hours to reach equilibrium,^28^ we hypothesized this chemical property would translate to time-dependent PPI stabilization. To investigate this hypothesis, we performed a td-FA assay were 14-3-3γ was titrated to a fluorescein-labelled Pin1 peptide in the presence or the absence of a stabilizer, and data was incrementally collected over 4 h. The resulting data was then processed using the workflow described in Fig. S2.† To compare non-covalent and covalent molecular glue stabilization, natural product fusicoccin-A (FC-A, Fig. 2B) was used as a non-covalent control stabilizer.^24,29,30^ To validate FC-A binds the same 14-3-3/Pin1 binding pocket, we crystalized the ternary complex (Fig. S1E–G†). Analysis of the crystal structure confirmed FC-A binds at the interface of the 14-3-3/Pin1 complex similarly to MG1, however, does not form a covalent bond with 14-3-3. Notably, 14-3-3*σ*ΔC, which is C-terminally truncated, was used as this mutant simplifies crystallization and is highly homologous to 14-3-3γ in structure and sequence (Table S1†). Analysis of the td-FA data showed the binary 14-3-3/Pin1 complex formed rapidly (<10 min, *K*_D_ = 30 ± 2 μM; Fig. 2C) and was constant over time (Fig. 2D). In the presence of FC-A rapid stabilization of the 14-3-3/Pin1 complex was also observed (apparent *K*_D_ (*K*_D_^app^) = 2.9 ± 0.2 μM), correlating to a ∼10-fold stabilization. In contrast, MG1 mediated stabilization proceeded *via* two phases. Initially, a rapid stabilization of the 14-3-3/Pin1 complex was observed (*K*_D_^app^ = 5.4 ± 1 μM at 10 min), followed by a slower second phase of stabilization taking 2–3 h to reach its maximum effect (*K*_D_^app^ = 1.5 ± 0.5 μM; Fig. 2C–D). This observation suggested the slow stabilization kinetics, compared with non-covalent FC-A, was a function of imine bond formation.

To further investigate the effects of imine-bond formation on PPI stabilization a td-FA assay was performed at varied pH (6.0–9.0) and the data was processed using the aforementioned workflow (Fig. S3†). The 14-3-3/Pin1 and the 14-3-3/Pin1/FC-A complexes were non-responsive to the pH variations under neutral and basic conditions (Fig. S4†). In contrast, the formation of the 14-3-3/Pin1/MG1 complex was highly responsive to pH (6.5–8.0) with a *K*_D_^app^ range of ∼5.2–0.6 μM (∼3.8–48-fold stabilization, Fig. 2E), in line with the pH-dependent behaviour of imine formation.^31,32^ Maximum MG1 stabilization of the 14-3-3/Pin1 complex was also observed after 2–3 h, dependent on pH (7.0–8.0) (Fig. 2F). While at pH 6.5 a constant *K*_D_^app^ of ∼5.2 μM was observed throughout the measurement, with no signs of the slower second phase of stabilization.

In addition to the pH, the reactivity of the aldehyde functional group is highly influenced by the electrochemical properties of the electrophilic small molecule. To investigate the electro-donating and electro-withdrawing properties of the molecular glue on PPI stabilization a panel of 2-*ortho* substituted benzaldehydes was used (Fig. 2G). Structural analysis of X-ray crystal structures of MG2–6 in complex with the 14-3-3*σ*ΔC/Pin1 complex showed all molecular glues shared a common binding mode to MG1 with an unambiguous electron density map for the ligand (Fig. S5†). Time-dependent FA experiments showed that electronegative substitutions enhance stabilization (MG4*versus*MG2) (Fig. 2H), with the trend remaining consistent over a range of pH values (Fig. S6†). However, stabilization was not solely driven by electronegativity, with 2-Cl (MG5) and 2-CF3 (MG6) analogues eliciting less stabilization than 2-Br (MG4). These results showed that 14-3-3/Pin1 stabilization is driven by an interplay of molecular recognition of the composite 14-3-3/Pin1 binding pocket and the reactivity of the aldehyde. This is best illustrated by MG2 that covalently binds 14-3-3/Pin1 yet did not stabilize the complex.

Next, we assessed if time-dependent molecular glue stabilization was unique to the ternary 14-3-3/Pin1/molecular glue complex. The previously published aldehyde-based molecular glue MG7 has also been shown to covalently ligate Lys122, and in turn stabilize the 14-3-3/p65 PPI (Fig. S7†).^33^ Using this ternary complex, we repeated the td-FA assay at various pH values (Fig. S8†). Analysis of the td-FA data using MG7, 14-3-3, and a p65 peptide showed a time- and pH-dependent molecular glue stabilization profile, similar to MG1. Interestingly, complete saturation of stabilization was observed at pH 8. Whilst MG1 and MG7, are not directly comparable given the scaffold and binding partner vary, these results provide valuable insight into aldehyde-based stabilization.

### MG1 preferential binds the preformed 14-3-3/Pin1 complex

**Fig. 3:**
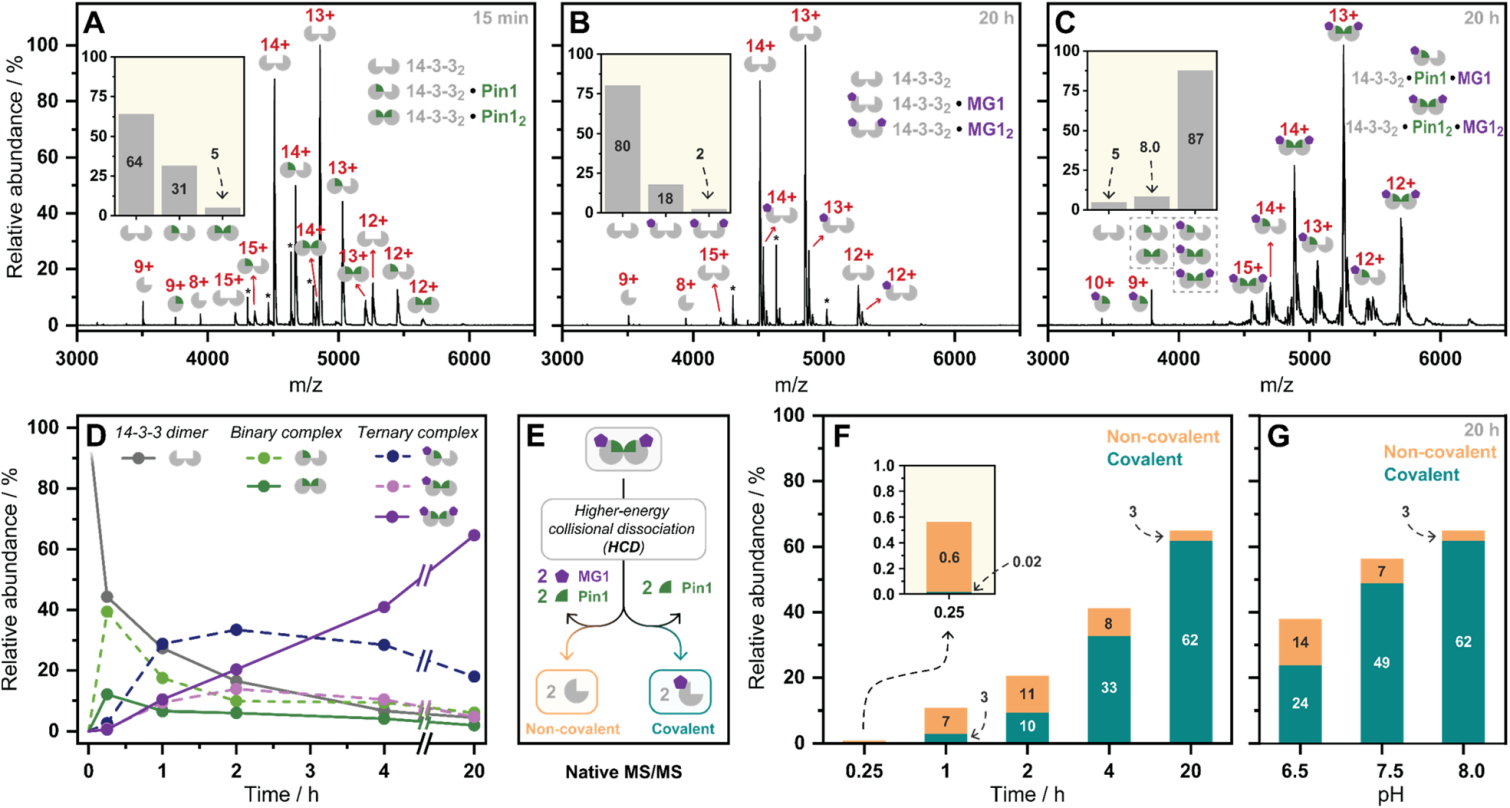
(A–C) Native mass spectra of the 14-3-3/Pin1, 14-3-3/MG1, and 14-3-3/Pin1/MG1 complexes. Inserts represent relative abundances of observed complexes. *Indicates protein contaminant. (D) Relative abundances of observed complexes over incubation time (pH 8.0). (E) Schematic representation of the native MS/MS experiment. (F) Relative abundance of non-covalent/covalent 14-3-3/Pin1/MG1 complex formation observed by native MS/MS over time (pH 8.0), (G) and over pH.

Having kinetically probed aldehyde-based molecular glue stabilization, native MS was subsequently employed to correlate stabilization to ternary complex formation. Specifically, by linking the observed profile to real-time quantification of abundances and stoichiometries of protein complexes. First, Pin1 was incubated with 14-3-3 (5 : 1 ratio) to confirm the formation of the binary complex without MG1 (Fig. 3A). Given 14-3-3 dimerizes in solution (Fig. S9†),^34^ we observed unbound 14-3-3 dimer (14-3-3_2_), single Pin1 bound 14-3-3 dimer (14-3-3_2_/Pin1), and double Pin1 bound 14-3-3 dimer (14-3-3_2_/Pin1_2_), with the apo 14-3-3 dimer (∼65%) dominating the mass spectrum at the set equivalents. MG1binding to 14-3-3 was then measured (5 : 1 ratio, Fig. 3B). Minimal MG1 bound 14-3-3 was observed after 20 h incubation, indicating that Pin1 is required for cooperative MG1 binding. In contrast, incubation of 14-3-3, Pin1 and MG1 (1 : 5 : 5 ratio) resulted in the ternary 14-3-3/Pin1/MG1 complexes as the dominant species after 20 h incubation (∼90%, Fig. 3C). Further analysis of these 14-3-3/Pin1/MG1 complexes showed the 14-3-3_2_/Pin1_2_/MG1_2_ complex was the major species (Fig. S10†), with minimal apo 14-3-3 dimer (≤5%) observed. Moreover, no complex was observed between Pin1 and MG1, indicating the specific nature of this stabilization (Fig. S11†). Taken together, these results indicate that the three components bind with high cooperativity wherein MG1 acts by ‘gluing’ 14-3-3 and Pin1 together, and Pin1 functions to template MG1.

Previously we have shown that MG1 induces conformational changes to the 14-3-3/Pin1 interface leading to cooperative complex formation.^17^ Given that molecular glue binding to 14-3-3 was partner protein dependent, we sought to investigate the kinetics of ternary complex assembly. Pin1, 14-3-3, and MG1 were incubated for 0.25, 1, 2, 4, and 20 h, and the abundance of each complex was quantified at each timepoint (Fig. S12†). After 15 min, the rapidly formed binary complexes (14-3-3_2_/Pin1 and 14-3-3_2_/Pin1_2_) were the dominating species, and trace amounts of stabilization (<5%) were observed (Fig. 3D). This was followed by the consumption of the binary complexes over the next 45 min with the 14-3-3_2_/Pin1_2_/MG1 complex being the dominant species after 2 h. MG1 induced stabilization continued until the most abundant species was the saturated 14-3-3_2_/Pin1_2_/MG1_2_ complex. Comparison of the native MS measurements at 15 min with and without MG1 showed enhanced consumption of apo 14-3-3, and an increase in both single and double Pin1 bound 14-3-3 dimer (Fig. S13†). This result suggests that MG1 accelerates 14-3-3/Pin1 complexation. Notably, double MG1 binding to the 14-3-3 dimer (14-3-3_2_/Pin1_2_/MG1_2_) in the native MS experiment resulted in an overall enhancement in the abundance of stabilized protein complexes, however, did not elicit an equivalent response to single MG1 binding (Fig. S14A and B†). This result indicates that the avidity of the total complex is further enhanced by a second molecular glue binding. Notably, at these stoichiometries (1 : 5 : 5 14-3-3 : Pin1 : MG1) there is limited 14-3-3, potentially explaining the lack of equivalent response upon double ligation. Interestingly, enhanced stabilization upon double MG1 binding was not observed in the FA experiments (Fig S14B†). This discrepancy may originate from concentration differences between the assay formats leading to different kinetics of ternary complex formation. Specifically, in the native MS experiments equimolar concentrations of MG1 and Pin1 were used, in contrast, in the td-FA experiment a 1000-fold excess of MG1 (relative to Pin1) was used, explaining the rapid stabilization observed in the FA experiments. This observation highlights the power of native MS as a technique to study molecular events, such as molecular glue binding.

Finally, a native MS/MS approach was developed to directly discriminate between non-covalent and covalent molecular glue binding in a time-deconvoluted manner. This approach isolated all complexes in the ion trap and then subjected these to a set higher-energy collision induced dissociation energy at which the non-covalent interactions between 14-3-3, Pin1, and MG1 were disrupted whilst conserving covalent interactions (Fig. 3E). This enabled the detection of covalently ligated 14-3-3/MG1, as demonstrated in a control experiment conducted after 2 h incubation of 14-3-3, Pin1 and MG1 (Fig. S15†). Consistent with the complex assembly seen in Fig. 3D, the ternary complex formation increased time-dependently (native MS/MS spectra shown in Fig. S16†), and positively correlated with MG1 ligation (Fig. 3F). For instance, at 15 min the 14-3-3/Pin1/MG1 species were predominantly non-covalent. After 45 min, the covalent ligation increased to ∼30%, with almost complete ligation at 20 h. These results showed that complex stabilization proceeded *via* a two-step MG1 ligation process.

To further dissect the role of imine bond formation, we performed native MS/MS experiments at varied pH. Analysis of the native MS spectra showed an increase in overall 14-3-3/Pin1/MG1 complex abundance upon increasing pH (Fig. S17†). Subsequent, analysis of the native MS/MS experiments showed pH-dependent ligation (20 h) (Fig. S18†), with ∼65% and ∼95% MG1 ligation at pH 6.5 and 8.0, respectively (Fig. 3G). A similar experiment at 15 min and varied pH confirmed the two-step ligation mechanism, with pH-independent non-covalent MG1 binding observed (Fig. S19†). Taken together the native MS/MS experiments correlated with the td-FA data, where stabilization was enhanced with increasing time and pH.

### Ternary complex formation proceeds *via* specific assembly order

**Fig. 4:**
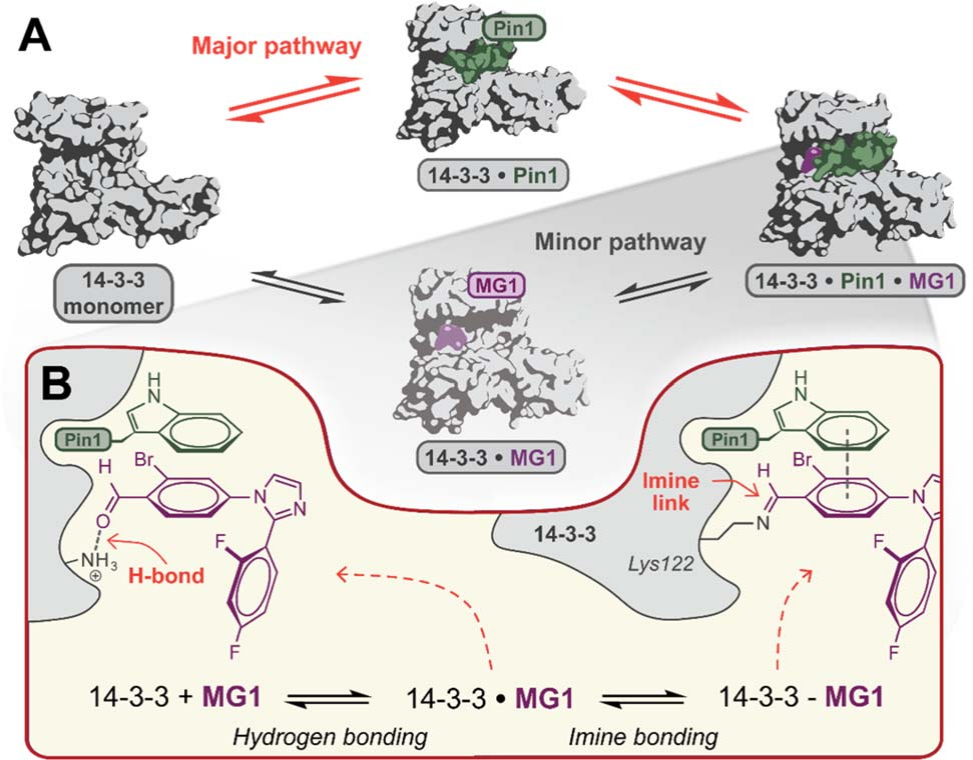
(A) Proposed hypothesis of the major pathway for 14-3-3/Pin1/MG1 complex assembly. (B) Proposed two-step molecular mechanism for MG1 binding to the 14-3-3/Pin1 complex.

Mechanistically, this study shows that MG1 has a weak affinity for apo 14-3-3, however, strongly binds the preformed 14-3-3/Pin1 complex (Fig. 3D and 4A). Further, MG1 stabilizes the 14-3-3/Pin1 PPI *via* a two-phase process where initially MG1 non-covalently binds and stabilizes the binary 14-3-3/Pin1 complex. As this initial phase is reached rapidly and is pH-independent, we propose that MG1 binds by hydrogen bonding between the ε-amino group of Lys122 and the aldehyde electrophile of MG1 (Fig. 4B). After the first phase, MG1 stabilization proceeds *via* a pH-responsive imine bond formation with enhanced cooperativity effects.

## Conclusions

In conclusion, we have unravelled the molecular mechanism of aldehyde-based molecular glue MG1 induced PPI stabilization. Ternary 14-3-3/Pin1/MG1 complex formation proceeds *via* a sequential order of assembly requiring preformation of the binary 14-3-3/Pin1 complex. This is scientifically significant as it experimentally probes existing multi-component equilibrium models^35^ in a time dimension. Further, it shows that high cooperativity can compensate for weak affinities of the molecular glue to the individual proteins. These results further debunk a misconception that affinity is the paramount parameter, showing a balanced view of molecular glue parameters are needed for molecular glue optimization. Additionally, our native MS/MS approach enabled the dissection of the non-covalent and covalent stabilization steps, both time- and pH-dependently. This revealed that MG1 stabilization proceeds *via* a two-step binding mechanism that consists of a rapid non-covalent association followed by slow covalent imine ligation. This shows that molecular glue ligation is regulated by a non-covalent cooperative binding event. Finally, this study provides a framework for the application of native MS to other molecular glues and more broadly covalent drug discovery. Importantly, this study is a conceptual step forward in drug discovery understanding, providing important biochemical information for future covalent molecular glue development.

## Data availability

The crystal structures elucidated in this manuscript have been deposited to the PDB (8C3C & 8C2G). All raw mass spectrometry data files are provided at https://doi.org/10.25500/edata.bham.00000951.

## Author contributions

CJAV, DFK, ACL and PJC. conceptualized and initiated the project. CJAV, and LD performed molecular glue synthesis and purification. DFK performed the native MS experiments. CJAV performed the FA and X-ray crystallography studies of unpublished compounds. RGD, LB scientifically consulted with the design of FA and native MS experiments. LB, ACL and PJC supervised the project and provided scientific guidance. CJAV and PJC wrote the manuscript with essential input and feedback from all other authors.

## Conflicts of interest

The authors declare the following competing financial interest: LB is a scientific co-founder of Ambagon Therapeutics.

## Supplementary Material

SC-014-D3SC01732J-s001
