# Supplementary material for: Tracking the mechanism of covalent molecular glue stabilization using native mass spectrometry
Source: Chem Sci. 2023 May 31;14(24):6756–62. doi: 10.1039/d3sc01732j (PMC10284121; doi:10.1039/d3sc01732j)
Supplement: SC-014-D3SC01732J-s001 [file SC-014-D3SC01732J-s001.pdf]

Electronic Supplementary Material (ESI) for Chemical Science.
This journal is © The Royal Society of Chemistry 2023

Supporting Information

Tracking the mechanism of covalent molecular glue stabilization using native mass spectrometry

Carlo J.A. Verhoef ‡[a], Danielle F. Kay ‡[b], Lars van Dijck[a], Richard G. Doveston[c], Luc Brunsveld[a], Aneika C. Leney*[b] and Peter J. Cossar*[a]

[a.] Department of Biomedical Engineering and Institute for Complex Molecular Systems, Eindhoven University of technology, Eindhoven, 5600 MB The Netherlands. E-mail: p.cossar@tue.nl
[b.] School of Biosciences, University of Birmingham, Birmingham, B15 2TT United Kingdom. E-mail: a.leney@bham.ac.uk
[c.] Leicester Institute of Structural and Chemical Biology and School of Chemistry, University of Leicester, Leicester, LE1 7RH United Kingdom

‡ These authors contributed equally.

Table of Contents

1. Supplementary Figures and Tables	3
2. Experimental Section – Protein expression and purification	17
3. Experimental Section – X-ray crystallography	18
4. Experimental Section – Fluorescence anisotropy assays	19
5. Experimental Section – Chemistry	23
6. Experimental Section – Native mass spectrometry	24
7. NMR data	25
8. LC-MS data	26
9. References	28

1. Supplementary Figures and Tables

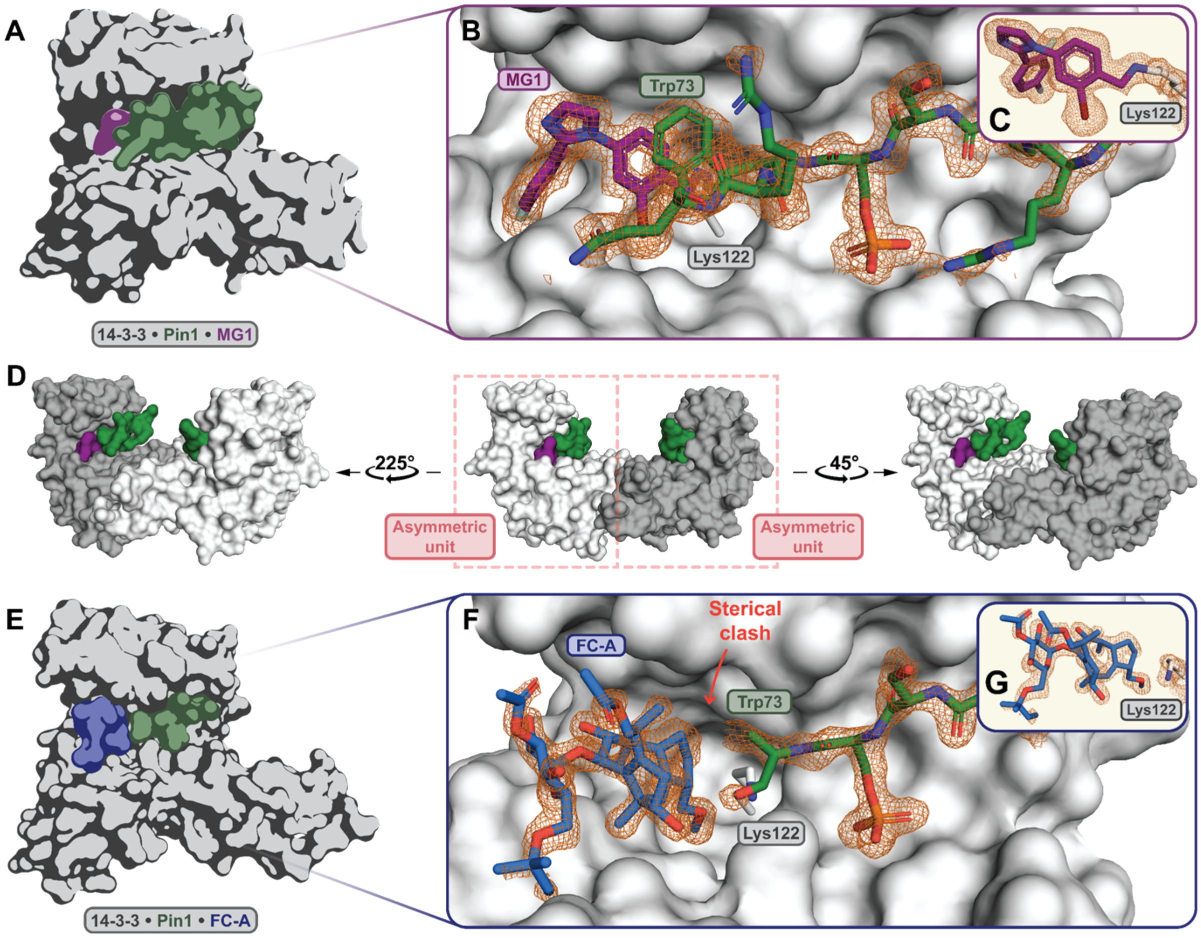

Fig. S1 Crystal structures show that MG1 and FC-A both stabilize the 14-3-3•Pin1 complex. MG1 stabilizes the 14-3-3/Pin1 interaction via an imine bond (A-C) The 14-3-3 dimer can bind two partner peptides and two stabilizers (D). The asymmetric unit of the 14-3-3_2•peptide$_2$•stabilizer$_2$ protein complex comprises of the 14-3-3•peptide•stabilizer complex (14-3-3_2•Pin1_2•**MG1**$_2$ shown as example; PDB code 7BFW, previous work[1]). **FC-A** non-covalently stabilizes the 14-3-3/Pin1 interaction (E-G; PDB code: 8C3C, structure elucidated in the current study). 14-3-3 (grey) is shown as surface model. Lys122 of 14-3-3 (grey), Pin1 (green), **MG1** (purple), and **FC-A** (blue) are shown as stick model. The $2F_o$-F_c electron density maps (orange) of Pin1, **MG1**, and **FC-A** are contoured at 1σ.

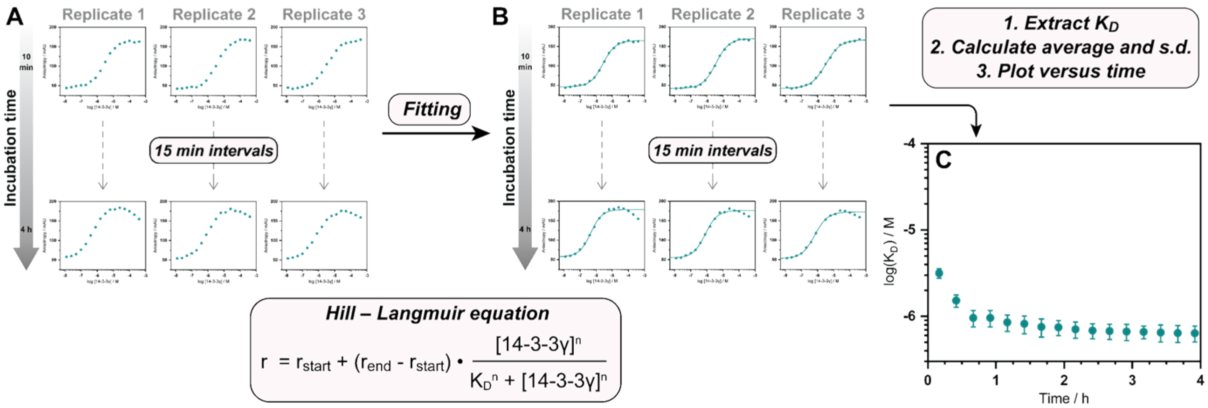

Fig. S2 Time dependent fluorescence anisotropy (td-FA) protein titration data processing. For each timepoint, FA data was measured in three replicate experiments and plotted versus the 14-3-3 concentration (A). For every replicate experiment, Hill-Langmuir equations were fitted to the FA data of each timepoint (B). The K_D values were extracted from the fitted functions, the average K_D values and standard deviations for every set of replicates were calculated (Supporting Tables S4-9), and plotted versus the incubation time (C).

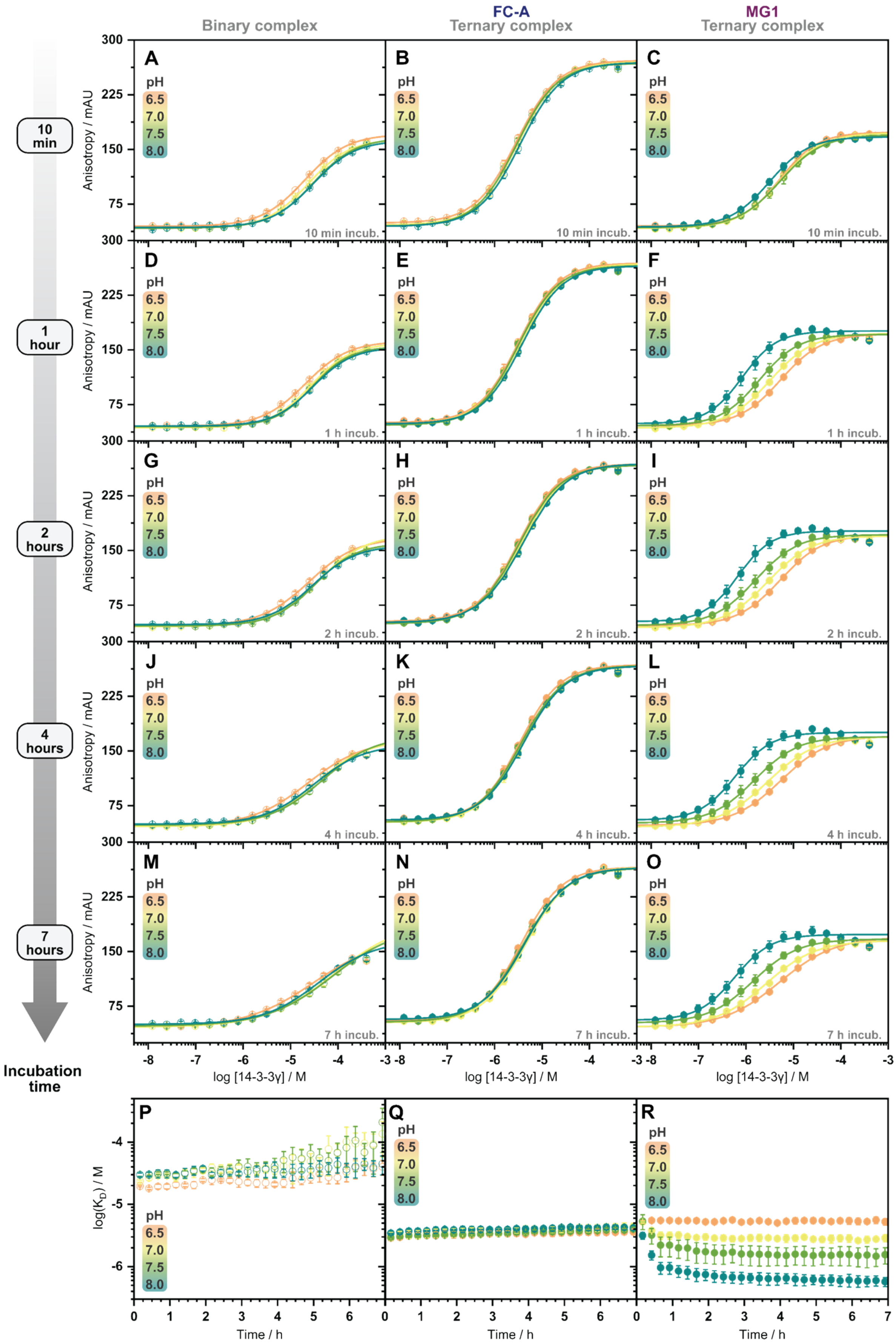

Fig. S3 Extended td-FA data for the binary, FC-A stabilized, and MG1 stabilized 14-3-3/Pin1 complexes. Raw td-FA data is shown for five timepoints (A-O), and the K_D profile is shown for the total experiment timespan (P-R). Open datapoints represent DMSO control (binary system); filled datapoints represent stabilized (ternary system).

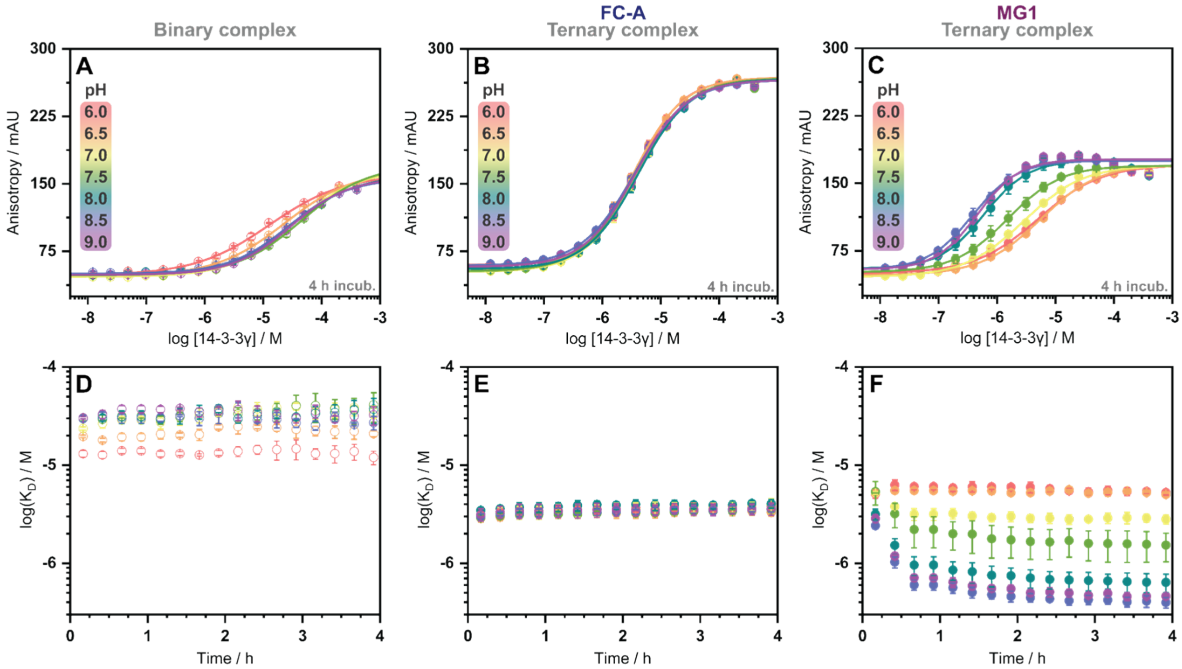

Fig. S4 td-FA data for an extended pH range for the binary, FC-A stabilized, and MG1 stabilized 14-3-3/Pin1 complexes. Raw td-FA data (4 hours incubation) is shown in the upper row (A-C), and the K_D profile is shown in the bottom row (D-F). Open datapoints represent DMSO control (binary system); filled datapoints represent stabilized (ternary system).

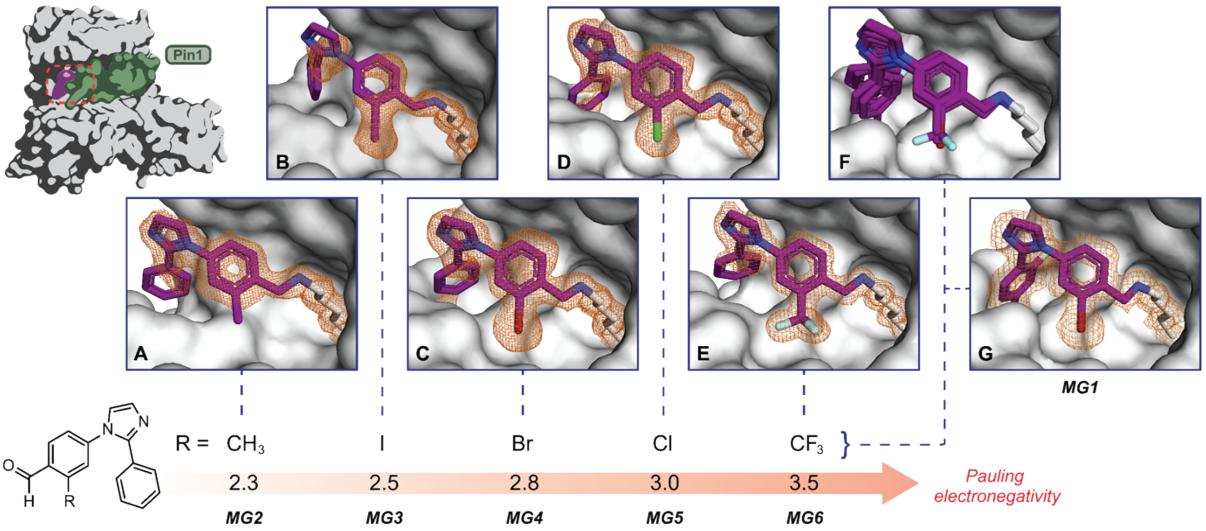

Fig. S5 The aldehyde compounds differing in *ortho* substituent stabilize the 14-3-3/Pin1 complex in a binding mode similar to MG1. 14-3-3σΔC/Pin1/compound crystal structures reveal that the members of the library bind highly similarly as **MG1** (A-G, with F representing an overlay). 14-3-3 (grey) is shown as surface model. Aldehyde compounds (purple) are shown as stick model. The $2F_o$-F_c electron density maps (orange) of the compounds are contoured at 1σ. For clarity, Pin1 is not shown in the panels. Crystal structures from PDB: code 7BGR (A), 7BDT (C), 7BDP (D), 7AZ1 (E), 7BFW (G). The 14-3-3σ•Pin1•**MG3** crystal structure was elucidated in the current study (PDB: code 8C2G). Pauling electronegativities from *Leroux et al.*[2]

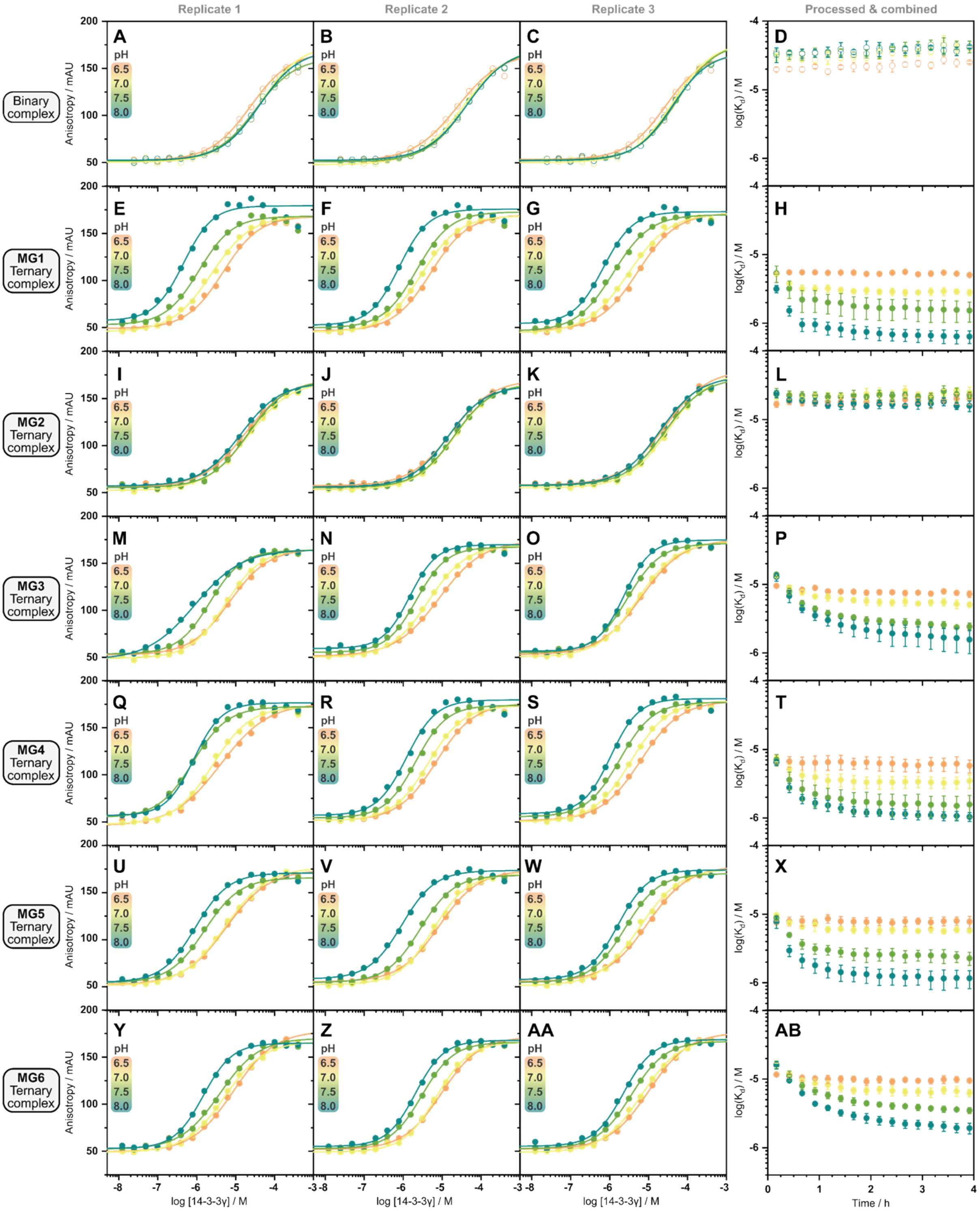

Fig. S6 td-FA data shows that the aldehyde compound library stabilizes the 14-3-3/Pin1 complex in two phases. The aldehyde compound library differing in *ortho* substituent was evaluated in td-FA assays, and showed a two phase stabilization effect similarly to **MG1**. Raw td-FA data (4 hours incubation) is shown in panels A-C, E-G, I-K, M-O, Q-S, U-W, Y-AA (three independent replicate datasets), and the K_D profile is shown in panels D, H, L, P, T, X, AB. Open datapoints represent DMSO control (binary system); filled datapoints represent stabilized (ternary system).

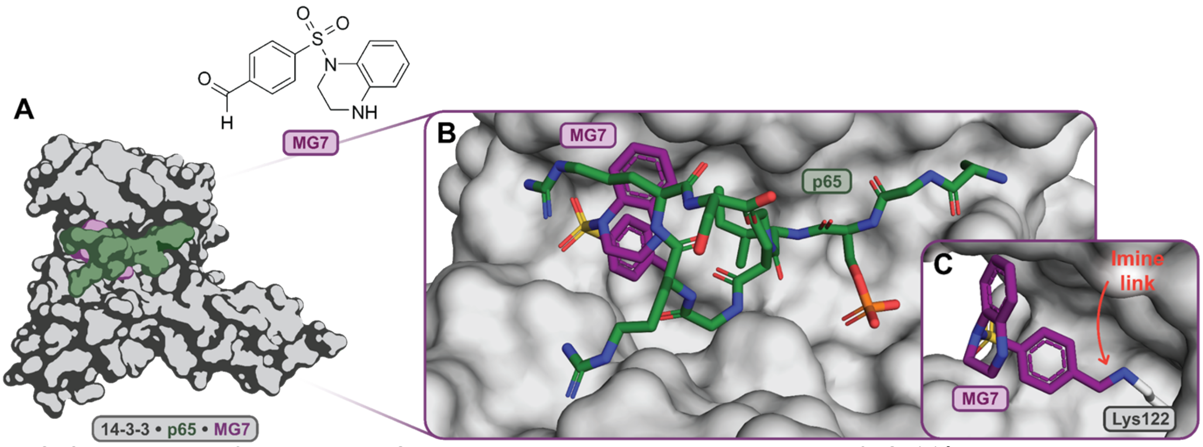

Fig. S7 Crystal structure of the 14-3-3•p65•MG7 complex. Ternary complex and chemical structure of **MG7** (A).[3] Binding groove containing the p65 peptide and **MG7** (B). Similarly to the previous aldehyde compounds, **MG7** binds via imine bonding to Lys122 of 14-3-3 (C). 14-3-3 (grey) is shown as surface model. p65 (green), and **MG7** (purple) are shown as stick model. Crystal structure from PDB: code 7BIW.

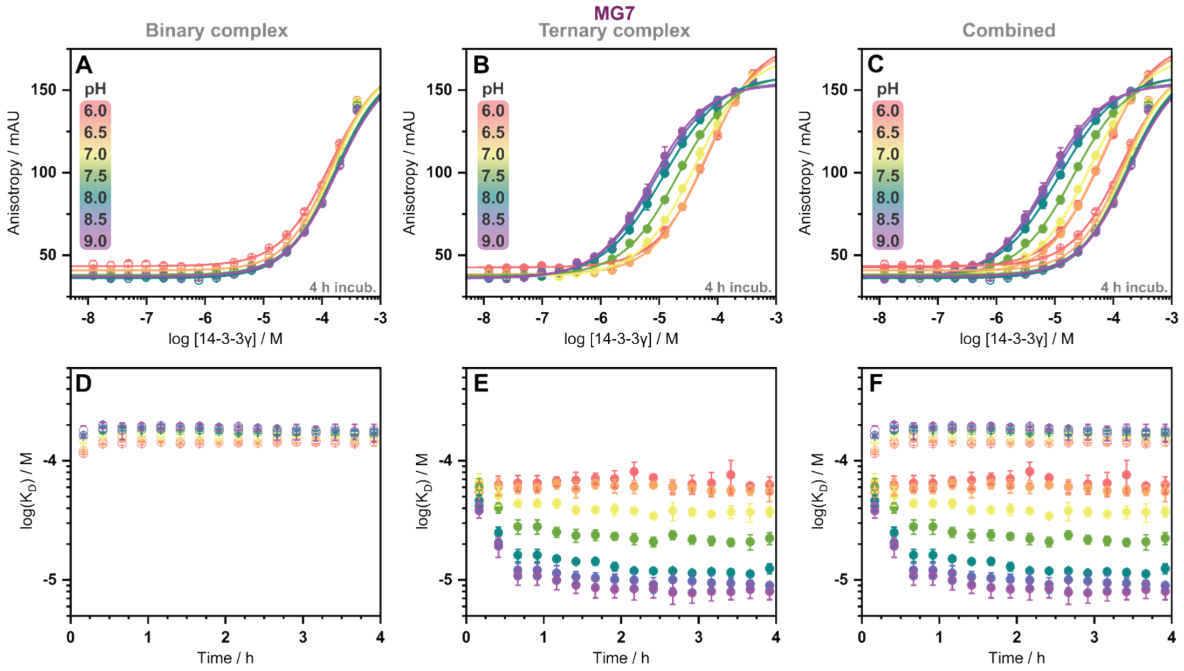

Fig. S8 td-FA data for an extended pH range for the binary and MG7 stabilized 14-3-3/p65 complexes. Raw td-FA data (4 hours incubation) is shown in the upper row (A-C), and the K_D profile is shown in the bottom row (D-F). Open datapoints represent DMSO control (binary system); filled datapoints represent stabilized (ternary system).

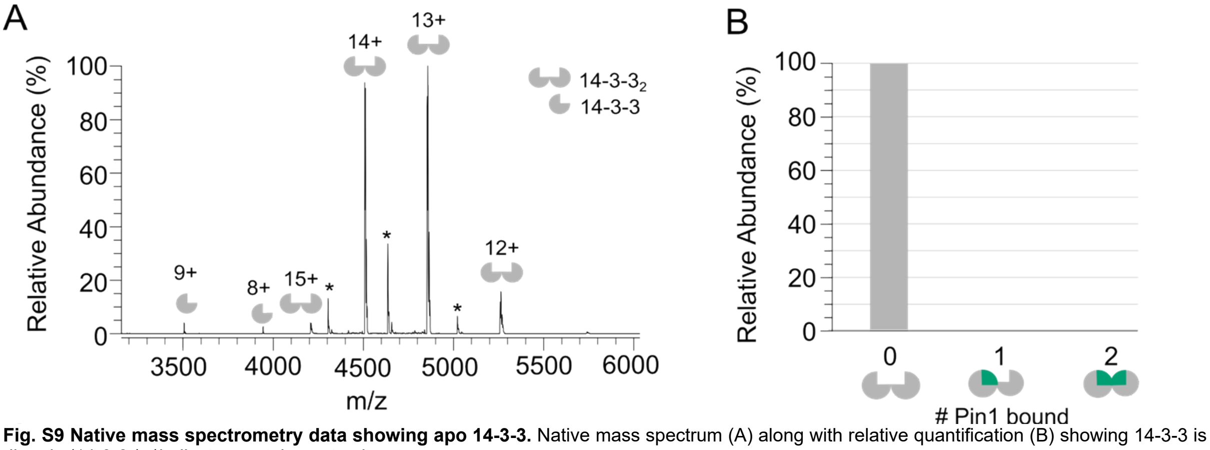

Fig. S9 Native mass spectrometry data showing apo 14-3-3. Native mass spectrum (A) along with relative quantification (B) showing 14-3-3 is dimeric (14-3-3$_2$). *indicates protein contaminant.

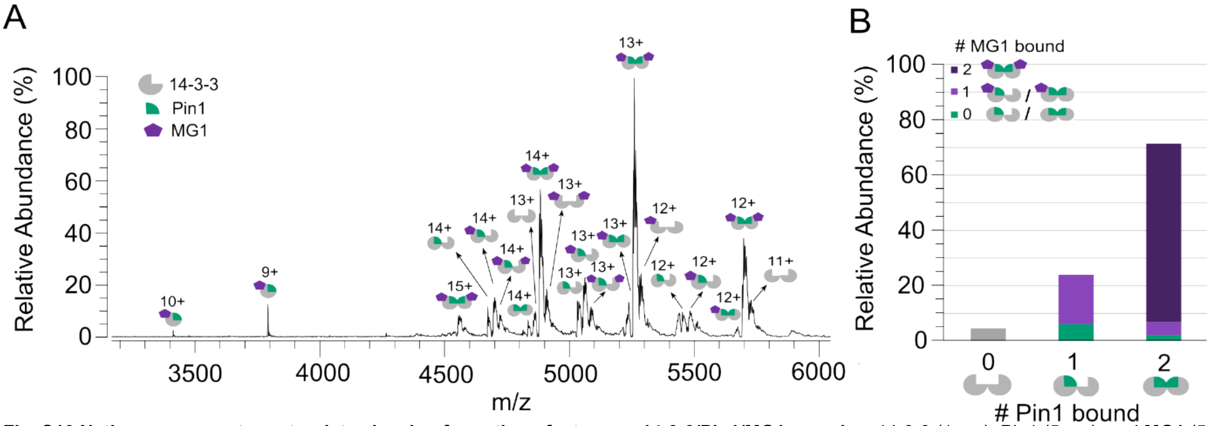

Fig. S10 Native mass spectrometry data showing formation of a ternary 14-3-3/Pin1/MG1 complex. 14-3-3 (1 eq.), Pin1 (5 eq.), and **MG1** (5 eq.) were incubated for 20 hours at pH 8.0 (A). 14-3-3$_2$/Pin1$_2$/**MG1**$_2$ is the most abundant complex with minimal unbound 14-3-3 and non-stabilized 14-3-3/Pin1 complexes observed (B).

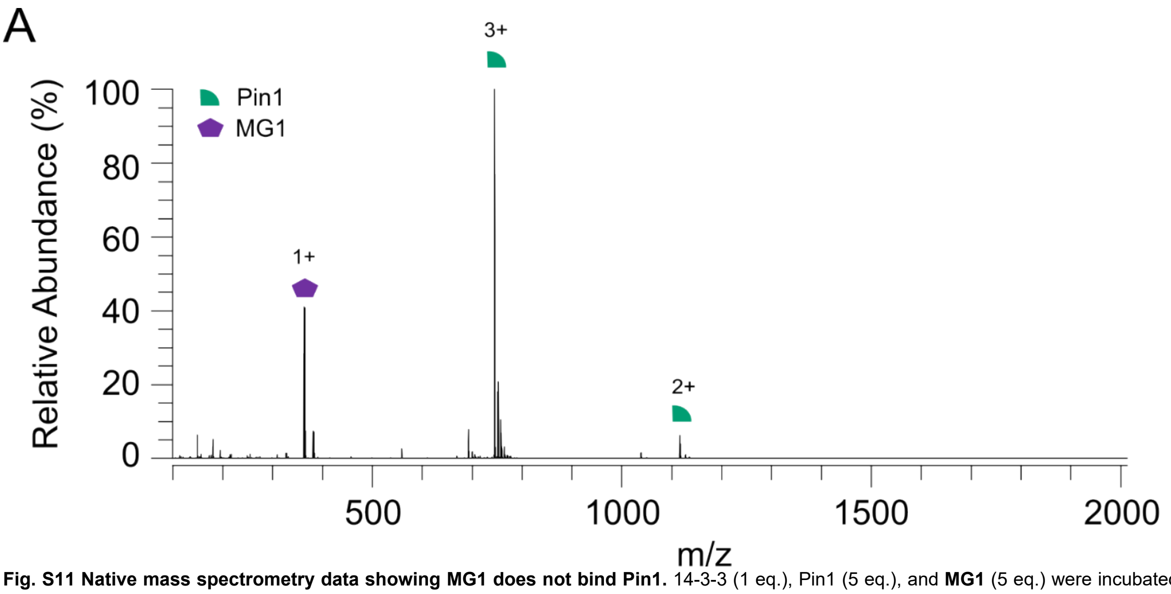

Fig. S11 Native mass spectrometry data showing MG1 does not bind Pin1. 14-3-3 (1 eq.), Pin1 (5 eq.), and **MG1** (5 eq.) were incubated for 20 hours at pH 8.0 prior to the measurement. A lower *m/z* range was set to monitor binding. No binding was observed between **MG1** and Pin1 in the low m/z region.

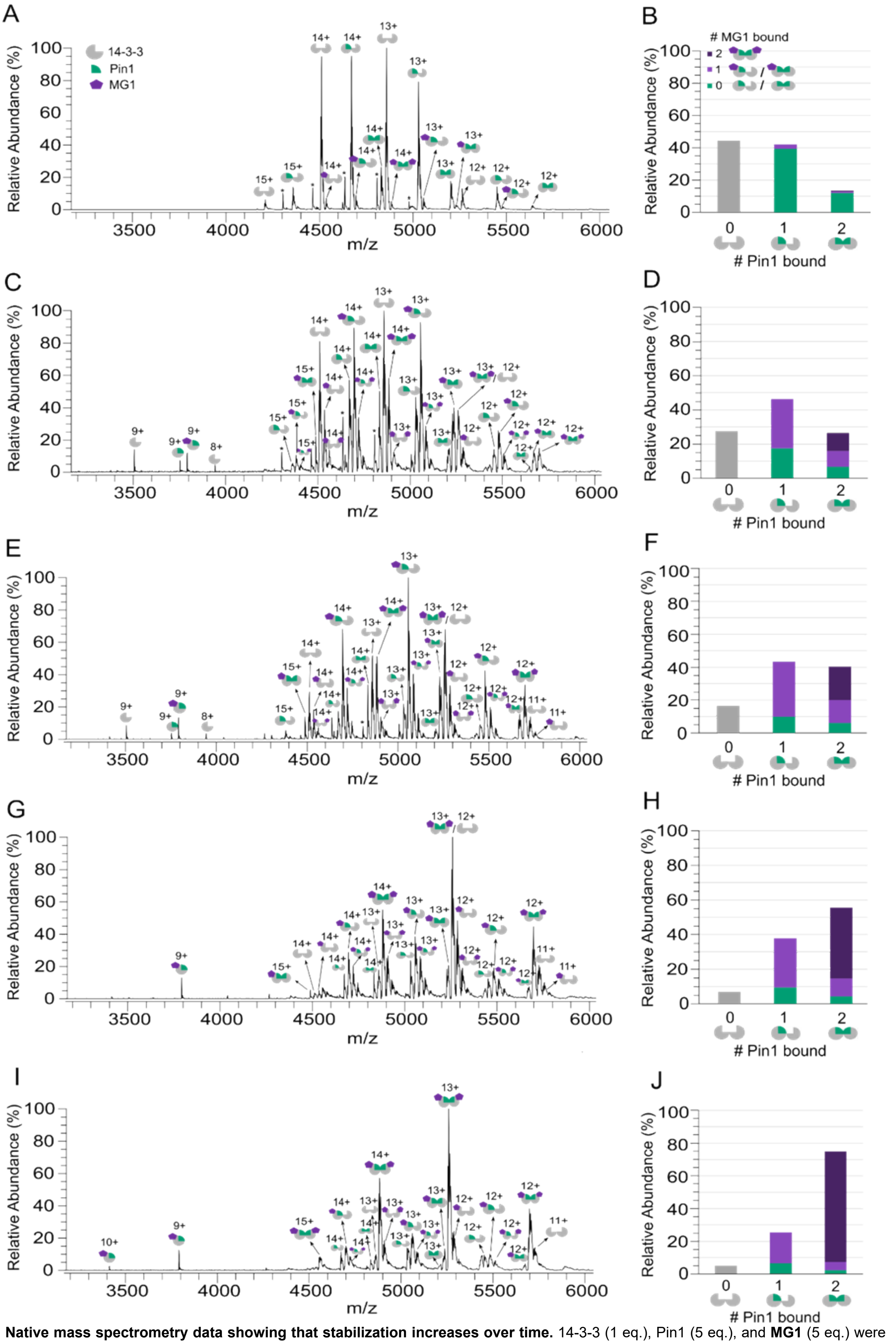

Fig. S12 Native mass spectrometry data showing that stabilization increases over time. 14-3-3 (1 eq.), Pin1 (5 eq.), and **MG1** (5 eq.) were incubated at pH 8.0 and native mass spectra acquired after 15 min (A,B), 1 hour (C,D), 2 hours (E,F), 4 hours (G,H), and 20 hours (I,J). Unbound 14-3-3_2 and 14-3-3_2/Pin1 were the most abundant complexes observed after 15 minutes (A, B). After 1 hour, the 14-3-3_2/Pin1/**MG1** complex appeared (C, D) with this dominating the spectrum after 2 hours (E, F). At 4 hours, the abundance of the 14-3-3_2/Pin1_2/**MG1**$_2$ complex started to increase (G, H) with this becoming the most dominant complex observed after 20 hours (I, J). Peaks labelled * correspond to a protein contaminant.

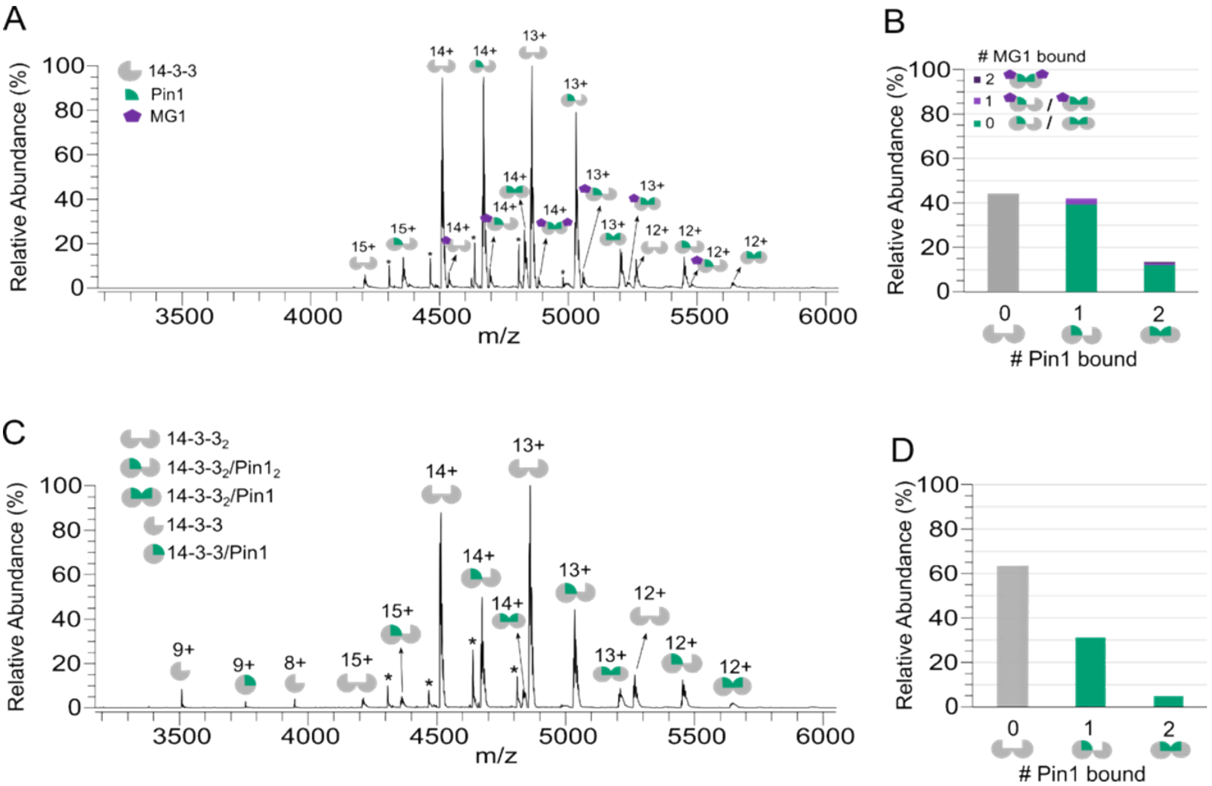

Fig. S13 Native mass spectrometry data showing that Pin1 binding is accelerated by MG1 after 15 min of incubation. 14-3-3 (1 eq.), Pin1 (5 eq.), and **MG1** (5 eq. in A,B; 0 eq. in C,D) were incubated at pH 8.0 and native mass spectra acquired after 15 min. Accelerated Pin1 binding to 14-3-3 is evident from the increased 14-3-3 consumption, and the formation of the stabilized species. Peaks labelled * correspond to a protein contaminant.

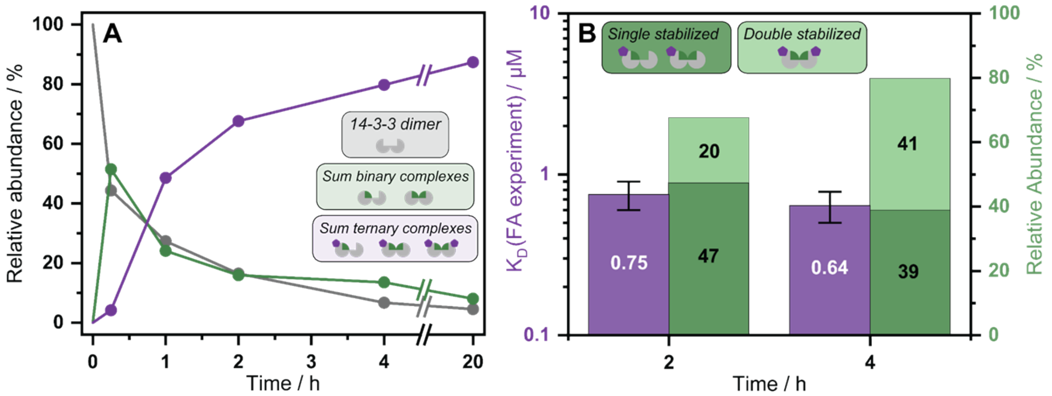

Fig. S14 Comparison between native MS and td-FA data. 14-3-3 (1 eq.), Pin1 (5 eq.), and **MG1** (5 eq.) were incubated at pH 8.0 and native mass spectra were acquired over time (A). The abundances of the indicated species were summed. Comparison of the K_D values measured by the td-FA experiments and abundances measured in native MS(/MS) at timepoints 2 and 4h. Both experiments were conducted at pH 8.0.

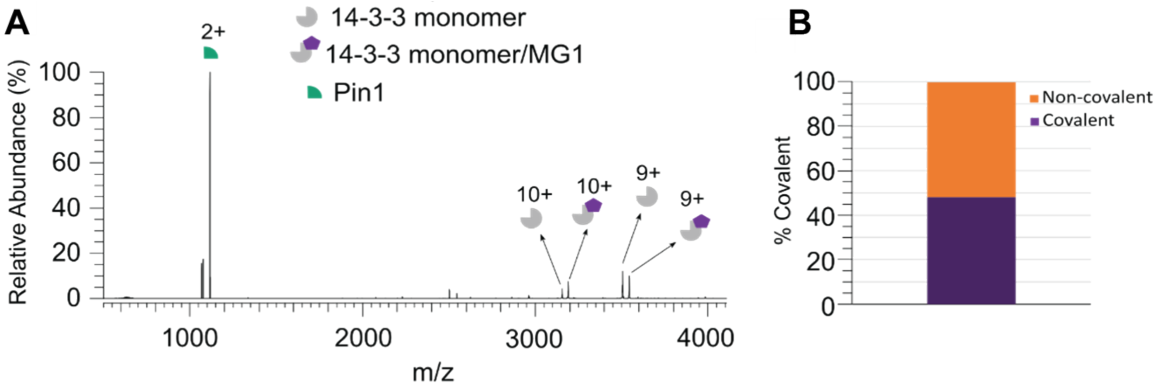

Fig. S15 Demonstration of the native MS/MS approach to quantify covalent ligation of MG1 to 14-3-3. 14-3-3 (1 eq.), Pin1 (5 eq.), and **MG1** (5 eq.) were incubated at pH 8.0 and a native MS/MS spectrum was acquired after 2 h (A). The native MS/MS spectrum shows that the 14-3-3/Pin1/**MG1** complex can be disrupted using higher-energy collisional dissociation energy to provide Pin1, 14-3-3 and 14-3-3/**MG1** complexes (B).

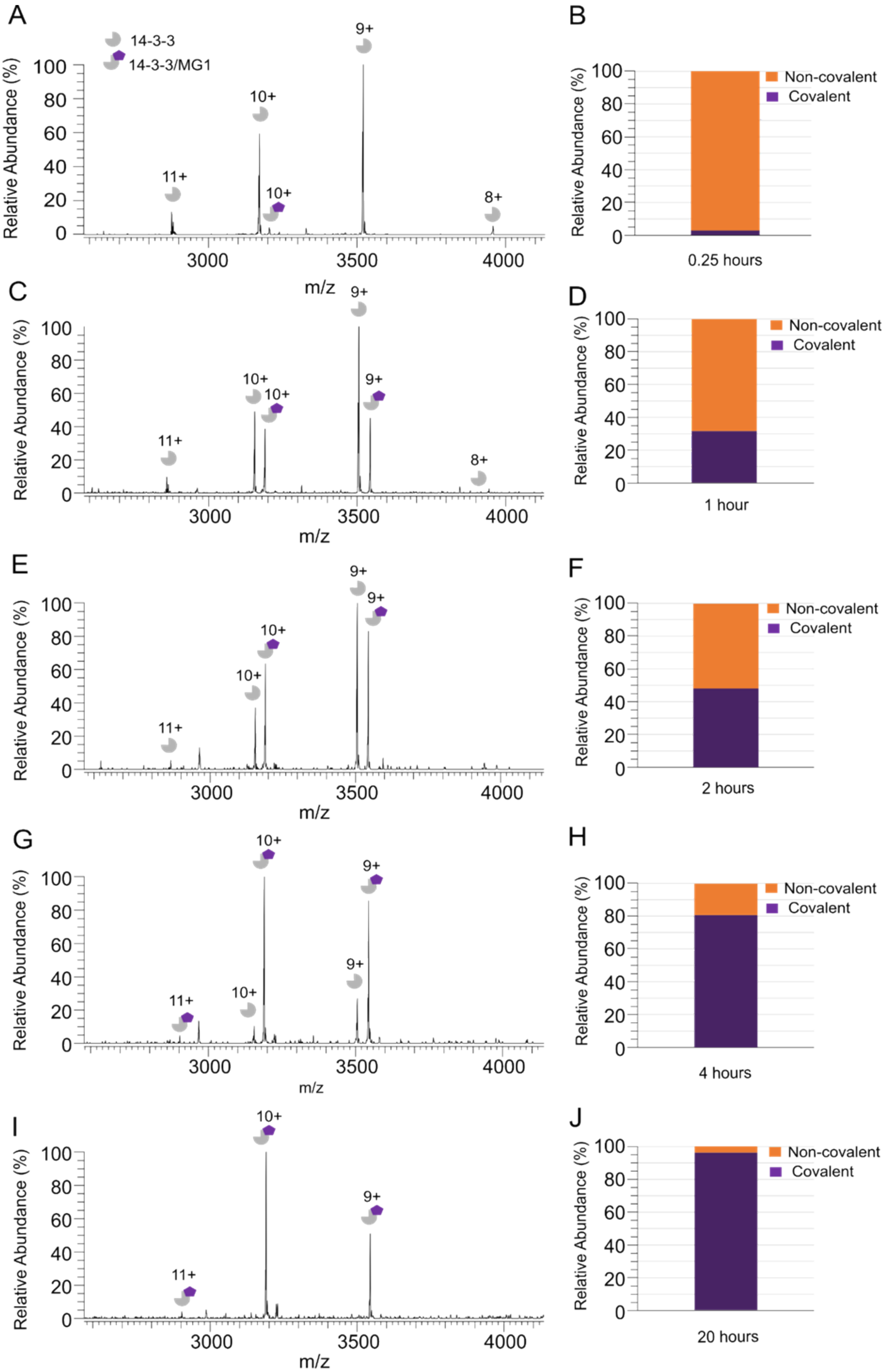

Fig. S16 Native MS/MS data showing covalent stabilization in a time-dependent manner. MS/MS spectra of released 14-3-3 monomer after 15 min (A, B), 1 hour (C, D), 2 hours (E, F), 4 hours (G, H) and 20 hours (I, J) incubation of 14-3-3 (1 eq.), Pin1 (5 eq.) and **MG1** (5 eq.). The abundance of 14-3-3/**MG1** increases over time, as well as the covalent/non-covalent ratio (shown in purple/orange).

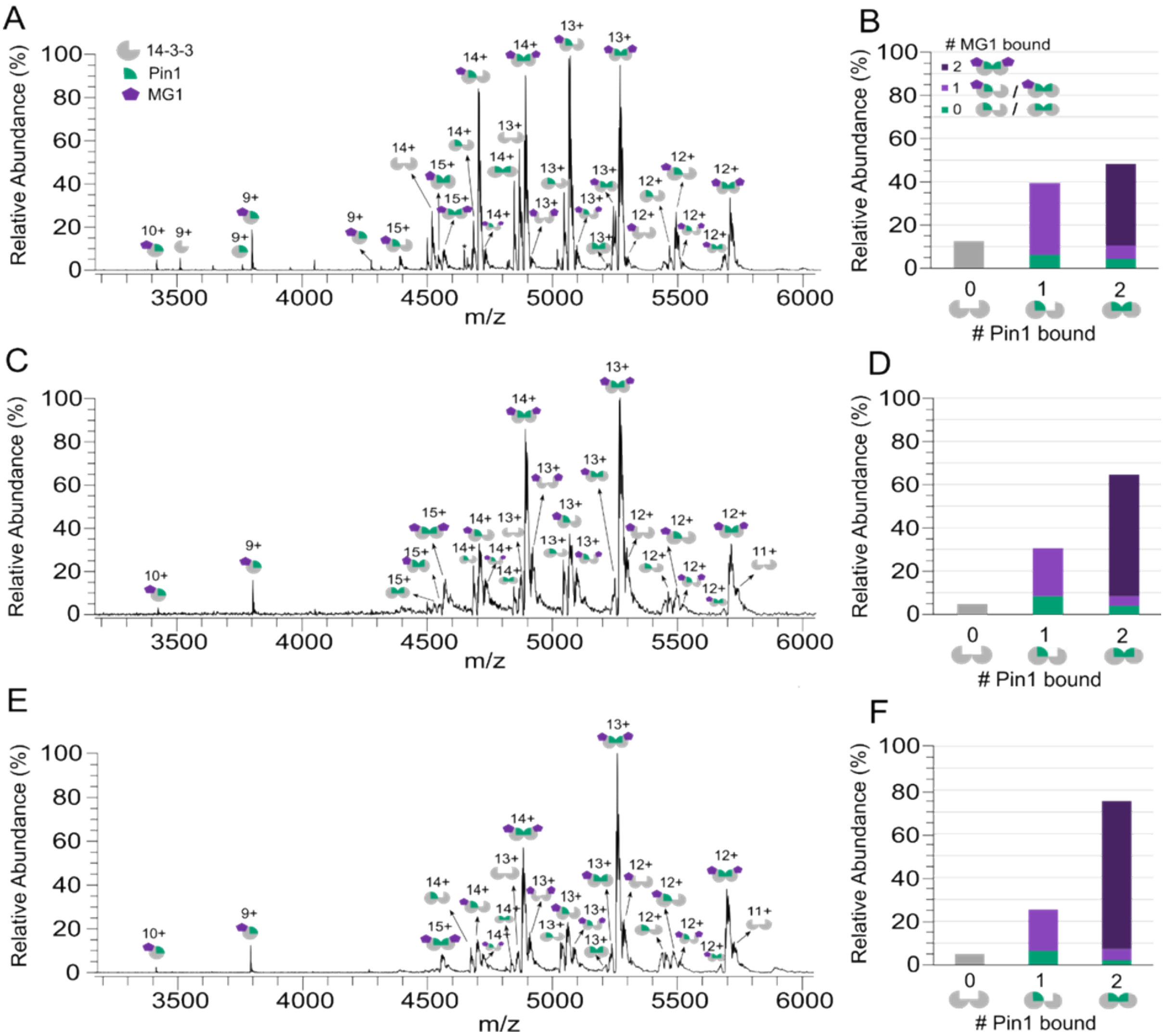

Fig. S17 Native mass spectrometry data showing covalent stabilization in a pH-dependent manner. Native mass spectra of mixtures containing 14-3-3 (1 eq.), Pin1 (5 eq.), and **MG1** (5 eq.) after 20 hours incubation at pH 6.5 (A, B), pH 7.5 (C, D) and pH 8.0 (E, F). At pH 6.5, 14-3-3$_2$/Pin1/**MG1** and 14-3-3$_2$/Pin1$_2$/**MG1**$_2$ are equally abundant. As the pH increases, a shift in equilibration is observed towards the saturated 14-3-3$_2$/Pin1$_2$/**MG1**$_2$ ternary complex.

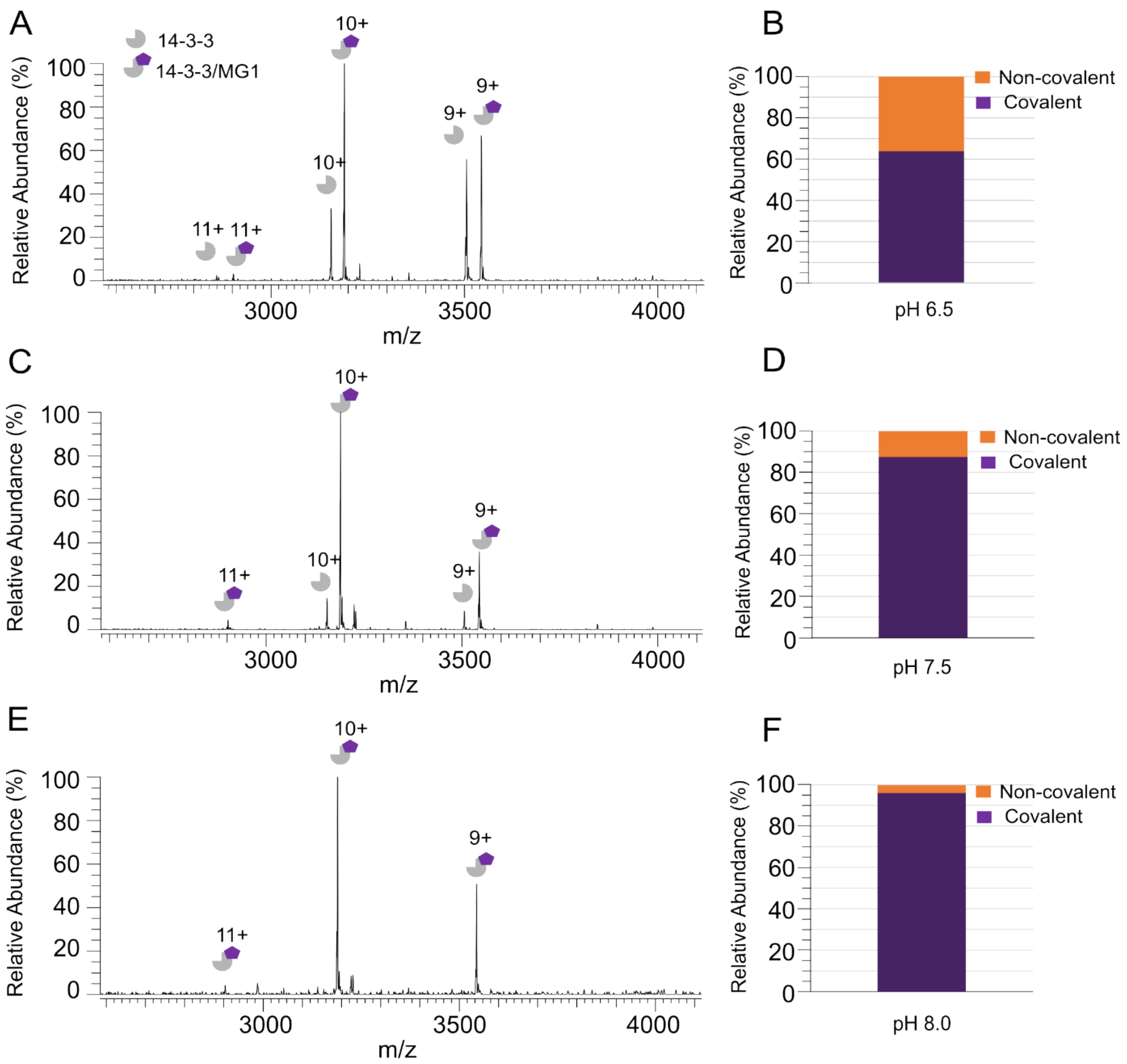

Fig. S18 Native MS/MS data showing covalent stabilization is pH-dependent. MS/MS spectra of released 14-3-3 monomer after 20 hours incubation of 14-3-3 (1 eq.), Pin1 (5 eq.) and **MG1** (5 eq.) at pH 6.5 (A, B), pH 7.5 (C, D), and pH 8.0 (E, F). The abundance of 14-3-3/**MG1** increases over the pH, as well as the covalent/noncovalent ratio (shown in purple/orange).

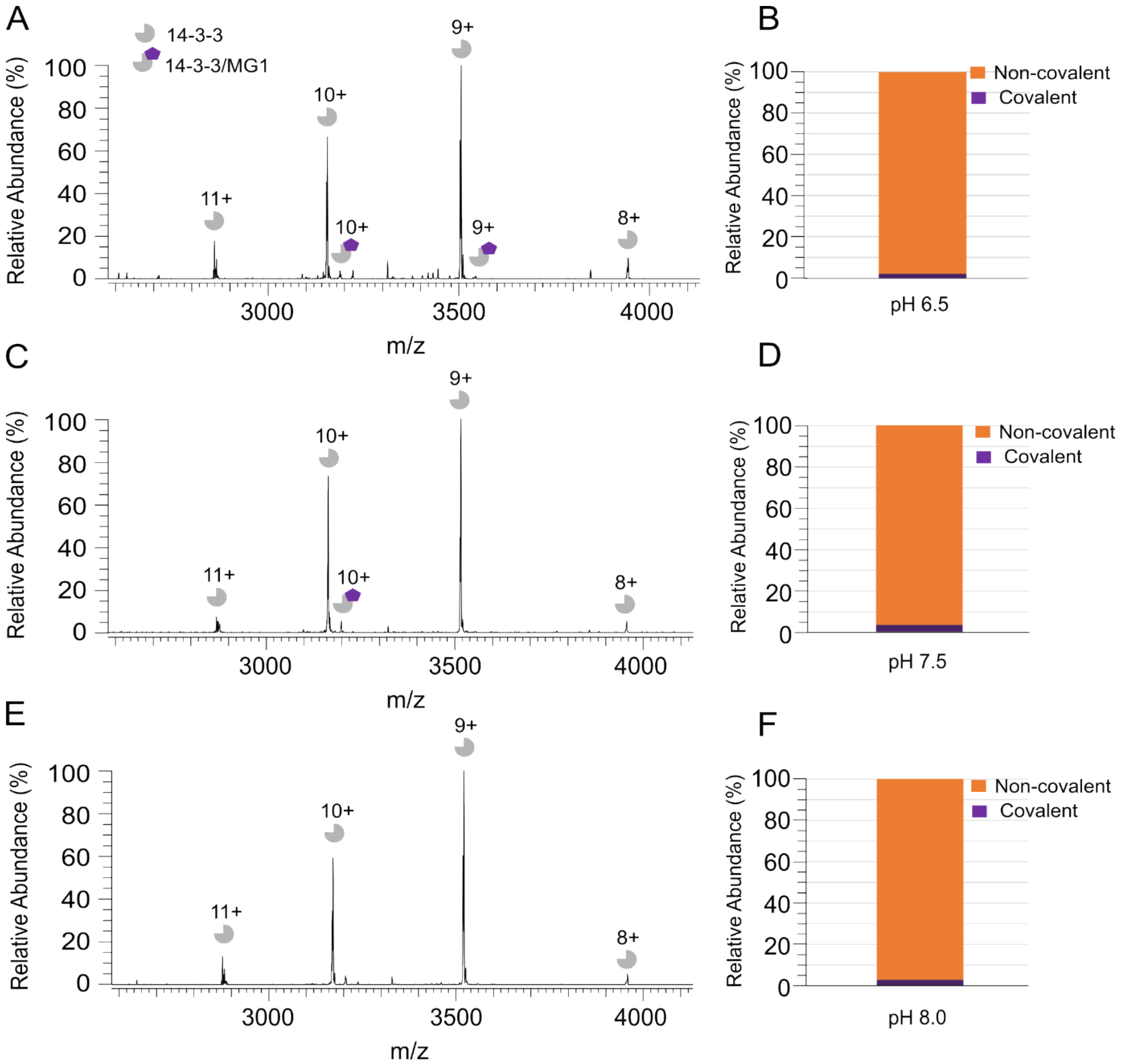

Fig. S19 Native MS/MS data showing non-covalent stabilization is pH-independent. MS/MS spectra of released 14-3-3 monomer after 15 mins incubation of 14-3-3 (1 eq.), Pin1 (5 eq.) and **MG1** (5 eq.) at pH 6.5 (A, B), pH 7.5 (C, D), and pH 8.0 (E, F). The abundance of 14-3-3/**MG1** is low in all cases, showing lack of covalent complex formation at early time points.

2. Experimental Section – Protein expression and purification

General materials

As previously reported in Cossar *et al.*, full length 14-3-3γ was used for FA assays (and native MS here), as Pin1 showed a higher affinity for this isoform than for 14-3-3σ.[1] 14-3-3σΔC was used for X-ray crystallography using previously reported conditions.[1] The two isoforms show high similarity (see protein sequence alignment below). Theoretical isoelectric points (pI) and molecular weights (MW) were calculated with the Expasy software.[4] Purity and exact mass of the proteins was determined using a high-resolution LC-MS system consisting of a Waters ACQUITY UPLC I-Class LC system coupled to a Xevo G2 Quadrupole Time of Flight (Q-TOF) mass spectrometer equipped with a Polaris C18A reverse phase column (2.0 x 100 mm, Agilent), using miliQ water with 0.1% formic acid (FA) and acetonitrile (ACN) with 0.1% FA, using a gradient of 5% to 100% ACN over 10 minutes. Sample concentration 0.1 mg/mL, injection volume 1 µL. Deconvolution of the m/z spectra was done using the MaxEnt1 algorithm (resolution 0.1 Da/channel) in the MassLynx software (version 4.2; SCN982). Mass spectra were centered (min peak width at half height 1, centroid top 80%) and errors were calculated using the MaxEnt Errors algorithm.

Table S1 Protein information.

Parameter	***Full length (FL) 14-3-3γ***	***14-3-3σΔC***
Average molecular weight (g/mole)	31543.02	26509.90
Theoretical pI	4.96	4.85
N-terminus	Purification tag and linker	Purification tag and linker removed by TEV proteolysis (required for crystallization)
C-terminus	Unmodified	Truncated after T231 (T236 in sequence alignment, required for crystallization)

Protein sequence alignment

Identities 161/239 (67%), positives 197/239 (82%), gaps 3/239 (1%), Lys122 is **underlined** in the sequences. Alignment adapted from the Basic Local Alignment Search Tool (BLAST)[5].

```
FL 14-3-3γ    1 SYYHHHHHHDYDIPTTENLYFQGAMGSMVDREQLVQKARLAEQAERYDDMAAAMKNVTELNEPLSNE  67
                                      GAMGSM +R  L+QKA+LAEQAERY+DMAA MK   E  E LS E
14-3-3σΔC                           1 GAMGSM-ERASLIQKAKLAEQAERYEDMAAFMKGAVEKGEELSCE  44

FL 14-3-3γ   68 ERNLLSVAYKNVVGARRSSWRVISSIEQKTSADGNEKKIEMVRAYREKIEKELEAVCQDVLSLLDNY 134
                ERNLLSVAYKNVVG +R++WRV+SSIEQK++ +G+E+K   VR YREK+E EL+ VC  VL LLD++
14-3-3σΔC    45 ERNLLSVAYKNVVGGQRAAWRVLSSIEQKSNEEGSEEKGPEVREYREKVETELQGVCDTVLGLLDSH 111

FL 14-3-3γ  135 LIKNCSETQYESKVFYLKMKGDYYRYLAEVATGEKRATVVESSEKAYSEAHEISKEHMQPTHPIRLG 201
                LIK   +   ES+VFYLKMKGDYYRYLAEVATG+ +  +++S+  AY EA +ISK+ M PT+PIRLG
14-3-3σΔC   112 LIKEAGDA--ESRVFYLKMKGDYYRYLAEVATGDDKKRIIDSARSAYQEAMDISKKEMPPTNPIRLG 176

FL 14-3-3γ  202 LALNYSVFYYEIQNAPEQACHLAKTAFDDAIAELDTLNEDSYKDSTLIMQLLRDNLTLWTSDQQDDD 268
                LALN+SVF+YEI N+PE+A  LAKT FD+A+A+L TL+EDSYKDSTLIMQLLRDNLTLWT
14-3-3σΔC   177 LALNFSVFHYEIANSPEEAISLAKTTFDEAMADLHTLSEDSYKDSTLIMQLLRDNLTLWT 236

FL 14-3-3γ  267 GGEGNN 274
```

Protein expression

Human 14-3-3 gamma was recombinantly expressed in BL21(DE3) cells using a pPROEX HTb vector. Cells were grown (37°C, 250 rpm) in prewarmed TB medium (supplemented with 1 mM $MgCl_2$, 0.1 mg/mL ampicillin) to an OD_{600}= 1.2. To induce expression 0.4mM isopropyl-β-d-thiogalactoside was added, followed by overnight shaking (18°C, 140 rpm). The cells were isolated by centrifugation (4°C, 8,600 rpm, 20 min) and resuspended in lysis buffer (50 mM HEPES, 300 mM NaCl, 12.5 mM imidazole, 5 mM $MgCl_2$, 2 mM β-mercaptoethanol, pH 8.0, filtered (0.2 µm)) supplemented with benzonase (10 µL/100 mL) and complete EDTA-free Protease Inhibitor Cocktail tablets (2 tablets/100 mL). The cells were lysed by homogenization twice (4°C, >15000 psi, C3 Emulsiflex-C3 homogenizer, Avestin), followed by centrifugation (4°C, 20,000 rpm, 30 min) to clear the lysate. For the Ni-NTA purification, a Ni-NTA Superflow cardridge (5 mL, 50 mg/mL binding capacity, 0.5 MPa, Qiagen, 5 mL column volume per liter culture) was washed with 5 column volumes (CVs) miliQ water, followed by 5 CVs elution buffer (50 mM HEPES, 300 mM NaCl, 250 mM imidazole, 2 mM β-mercaptoethanol, pH 8.0, filtered (0.2 µm)), and equilibrated with 10 CVs wash buffer (50 mM HEPES, 300 mM NaCl, 25 mM imidazole, 2 mM β-mercaptoethanol, pH 8.0, filtered (0.2 µm)) at 5 mL/min. The lysate supernatant was loaded onto the column (3 mL/min), followed by 10 CVs wash buffer (5 mL/min). The protein was eluted using 9.5 CVs elution buffer (5 mL/min), and collected into 0.5 ; 2 ; 1 ; 3 ; 3 CVs fractions. Fractions containing 14-3-3γ were combined and dialyzed twice (4°C, 10k SnakeSkin dialysis bag, 4 hour and overnight dialysis) into storage buffer (25 mM HEPES, 100 mM NaCl, 10 mM $MgCl_2$, 500 µM tris(2-carboxyethyl)phosphine (TCEP), pH 8.0, filtered (0.2 µm)) to remove excess imidazole. The protein was concentrated to 74.8 mg/mL (2370 µM) (4°C, 10k Amicon spin filters, 4000 rpm), transferred into 50 µL aliquots, flash-frozen, and stored at -70 °C. The molecular weight of the protein was confirmed using Q-TOF LC-MS analysis, calculated mass 31543.0; found 31542.8.

Human 14-3-3 sigma truncated after T231 (14-3-3σΔC) was recombinantly expressed in BL21(DE3) cells using a pPROEX HTb vector. Cells were cultured, induced and lysed, and 14-3-3σ was purified (Ni-NTA) similar to 14-3-3γ expression. Fractions containing 14-3-3σ were combined and dialyzed (4°C, 10k SnakeSkin dialysis bag, overnight) into dialysis buffer (25 mM HEPES, 200 mM NaCl, 10 mM $MgCl_2$, 2 mM β-mercaptoethanol, pH 8.0, filtered (0.2 µm))

supplemented with TEV protease (1 mg TEV protease / 10 mg 14-3-3σ) to remove the purification tag. For the anti Ni-NTA purification, a Qiagen Ni-NTA Superflow cardridge (5 mL, 50 mg/mL binding capacity, 0.5 MPa, Qiagen, 5 mL column volume per liter culture) was washed with 5 column volumes (CVs) miliQ water, followed by 5 CVs elution buffer (50 mM HEPES, 300 mM NaCl, 250 mM imidazole, 2 mM β-mercaptoethanol, pH 8.0, filtered (0.2 µm)), and equilibrated with 10 CVs wash buffer (50 mM HEPES, 300 mM NaCl, 25 mM imidazole, 2 mM β-mercaptoethanol, pH 8.0, filtered (0.2 µm)) at 5 mL/min. The dialyzed 14-3-3σΔC protein was loaded onto the column (3 mL/min), followed by wash buffer (~4 CVs, 5 mL/min) until no protein absorbance could be detected in the fractions. Fractions containing 14-3-3σΔC were combined, concentrated to 20 mg/mL (4°C, 3k Amicon spin filters, 3500 rpm), and filtered (0.2 µm). The protein was further purified by Size Exclusion Chromatography (SEC): the column (Superdex 75 pg 16/60 size exclusion column (GE Life Sciences), CV=120 mL, 1 mL/min) was equilibrated with 1.2 CV SEC buffer (25 mM HEPES, 100 mM NaCl, 10 mM $MgCl_2$, 500 µM tris(2-carboxyethyl)phosphine (TCEP), pH 8.0, filtered (0.2 µm)), the 14-3-3σΔC sample was loaded, and eluted using 1.2 CV SEC buffer. Fractions containing 14-3-3σΔC were combined and concentrated to 55.15 mg/mL (2080 µM) (4°C, 10k Amicon spin filters, 4000 rpm), transferred into 50 µL aliquots, flash-frozen, and stored at -70 °C. The molecular weight of the protein was confirmed using Q-TOF LC-MS analysis, calculated mass 26509.9; found 26509.7.

3. Experimental Section – X-ray crystallography

Table S2 Crystal structures overview.

Protein complex	***PDB code***	***Preparation***
14-3-3σΔC • Pin1 • **FC-A**	8C3C	*This manuscript*
14-3-3σΔC • Pin1 • **MG1**	7BFW	*Cossar et al.*[1]
14-3-3σΔC • Pin1 • **MG2**	7BGR	*Cossar et al.*[1]
14-3-3σΔC • Pin1 • **MG3**	8C2G	*This manuscript*
14-3-3σΔC • Pin1 • **MG4**	7BDT	*Cossar et al.*[1]
14-3-3σΔC • Pin1 • **MG5**	7BDP	*Cossar et al.*[1]
14-3-3σΔC • Pin1 • **MG6**	7AZ1	*Cossar et al.*[1]
14-3-3σΔC • p65 • **MG7**	7BIW	*Wolter et al.*[3]

Binary protein complexes were prepared by mixing 12 mg/mL 14-3-3σΔC (truncated after T231 to reduce flexibility) in a 1:2 ratio with Pin1 17mer peptide (LVKHSQSRRPSpSWRQEK, N terminal acetylated, Genscript)[1] in complexation buffer (20 mM HEPES pH 7.5, 2 mM $MgCl_2$, 2 mM β-mercaptoethanol, filtered (0.2 µm)), followed by overnight incubation at 4 °C. The binary protein complexes were crystallized in a hanging drop set up (4 °C), whereby the complexation solution (250 nL) was mixed with precipitation buffer (250 nL; 95 mM HEPES pH 7.1, 28% PEG400, 190 mM $CaCl_2$, 5% glycerol, filtered (0.2 µm)). The crystals grew within two weeks, and were subsequently soaked for 7 days with **FC-A** or **MG3** (final concentration 10 mM, final DMSO ≤ 1%) to form the 14-3-3σΔC/Pin1/**FC-A** and 14-3-3σΔC/Pin1/**MG3** crystals, respectively. Crystals were directly flash-frozen in liquid nitrogen after fishing and data acquisition took place at ID23-1. Settings were 1440 images, 0.25°/ image, 15% transmission and 10 ms exposure time. autoPROC (version 1.1.7) was used to index and integrate the diffraction data.[6] The data was further processed using the CCP4i2 suite (version 8.0.002).[7] Scaling was performed using AIMLESS.[8,9] MolRep[10,11] was used for phasing, using the binary 14-3-3σΔC/Pin1 structure (PDB code: 7AOG) as a template. Using the SMILES code of each compound, a three dimensional structure of the compound was generated using AceDRG[12], which was thereafter build in the 14-3-3σΔC/Pin1 structure utilizing the F_o-F_c and $2F_o$-F_c electron density maps in COOT (version 0.9.8.1).[13] Alternating cycles of model improvement and refinement were performed using COOT, REFMAC (version 5)[14,15], and phenix.refine (phenix software suite, version 1.20.1)[16,17]. Figures were generated with PyMOL (version 2.5.2). $2F_o$-F_c electron density maps were contoured at 1σ.

Table S3 Data collection and refinement statistics of crystal structures. Statistics for the highest-resolution shell are shown in parentheses.

**PDB code**	**8C3C**	**8C2G**
Protein	14-3-3σΔC	14-3-3σΔC
Peptide	Pin1 17mer	Pin1 17mer
Compound	**FC-A**	**MG3**
Beamline	ESRF ID23-1	ESRF ID23-1
Data collection		
Wavelength (Å)	0.972425	0.972425
Space group	C 2 2 21	C 2 2 21
Cell dimensions<br>a ; b ; c (Å)<br>α ; β ; γ (°)	 82.85 ; 111.54 ; 62.54<br>90.00 ; 90.00 ; 90.00	 82.16 ; 111.82 ; 62.68<br>90.00 ; 90.00 ; 90.00
Resolution (Å)	66.51 – 1.60 (1.63 – 1.60)	62.68 – 1.60 (1.63 – 1.60)
I / σ(I)	30.1 (7.1)	41.5 (11.8)
Completeness (%)	100.00 (100.00)	100.0 (100.0)
Redundancy	13.3 (13.4)	13.3 (13.4)
$CC_{1/2}$	0.999 (0.987)	0.999 (0.991)
Refinement		
No. reflections	38582	38452
R_{work}/R_{free}	0.192/0.200	0.179/ 0.186
No. atoms<br>Macromolecules	 1925	 1880

Ligands<br>Water	49<br>214	21<br>235
B-factors<br>Macromolecules<br>Ligands<br>Water	 22.86<br>36.42<br>31.98	 18.68<br>58.82<br>30.64
R.M.S. deviations<br>Bond lengths (Å)<br>Bond angles (°)	 0.012<br>1.425	 0.013<br>1.552
Ramachandran<br>favored (%)<br>outliers (%)	 98.25<br>0.00	 98.17<br>0.00
Clashscore	1.81	2.69

4. Experimental Section – Fluorescence anisotropy assays

FA assays were conducted using FITC labeled Pin1 17mer peptide (FITC-Ahx-LVKHSQSRRPSpSWRQEK, Genscript)[1] or FITC labeled p65 13mer peptide (FITC-βAla-EGRSAGpSIPGRRS, Genscript)[3], and 14-3-3γ in the absence (DMSO control) and presence of compound. Peptide stock solutions (5 μM) were prepared by the addition of miliQ water, compound stock solutions (20 mM) were prepared by the addition of DMSO. Both stocks were stored at -30 ºC. For protein titrations, 100 nM FITC labeled Pin1 peptide, 100 μM compound, and a variable concentration of 14-3-3γ (titrant, 400 μM starting concentration) were used. All FA assays were conducted using freshly prepared FA buffer (10 mM HEPES, 150 mM NaCl, 0.1% Tween20, 1 mg/mL BSA, variable pH (HCl, NaOH) measured using a laboratory glass electrode pH meter) in Corning 384 well plates (black, round bottom, low binding surface, 10 μL sample per well). Titrations were performed using a Thermo Scientific E1-ClipTip autopipette (1:1 dilution series). Measurements were performed directly after plate preparation, using a Tecan Spark plate reader (25 °C; λ_{ex} 485 ± 20 nm; λ_{em} 535 ± 25 nm; dichroic 510 mirror; 30 flashes; 40 μs integration time; 0 μs lag time, 1 ms settle time; gain 49; Z-position: calculated from well, 15 min measurement intervals). Wells containing FITC labeled Pin1 peptide in FA buffer were used to calculate the G-factor. The data was plotted using Origin 2020 software. Using the same software, sigmoidal functions were fitted to the data using the following formula: anisotropy = start + (end - start)·(x^n) / (k^n + x^n); start = bottom asymptote; end = top asymptote; x = titrant concentration; k = K_D; n = Hill coefficient. The datapoint with the highest titrant concentration was always excluded from the fitting procedure, as this datapoint suffers from a buffer mismatch. All results are based on three independent experiments from which the average and standard deviation for each fitted K_D value (Tables S4-9) were calculated using Microsoft Excel. For the stabilized (ternary) 14-3-3/Pin1 complexes, the fitted K_D values represent apparent K_D values (K_D^{app}).

Table S4 Kinetic K_D data for the formation of the binary 14-3-3/Pin1 complex. Average K_D values (av.), and corresponding standard deviations (s.d.) are given in μM.

Incub. Time (h)	Binary 14-3-3/Pin1 complex													
	pH 6.0		pH 6.5		pH 7.0		pH 7.5		pH 8.0		pH 8.5		pH 9.0	
	av.	s.d.	av.	s.d.	av.	s.d.	av.	s.d.	av.	s.d.	av.	s.d.	av.	s.d.
0.17	13.06	1.00	19.65	0.58	23.65	0.85	29.97	1.71	29.79	2.01	29.84	2.20	30.37	0.76
0.42	12.70	1.02	17.99	0.79	26.68	2.68	29.35	0.23	30.35	2.25	32.25	2.50	33.02	3.22
0.67	13.99	0.83	19.35	1.68	28.89	1.92	31.65	1.52	31.10	4.58	29.88	3.06	37.30	3.16
0.92	13.99	0.83	19.35	1.68	28.89	1.92	31.65	1.52	31.10	4.58	29.88	3.06	37.30	3.16
1.17	13.00	0.83	20.74	2.17	27.31	3.90	28.79	2.64	29.82	2.82	30.47	2.63	37.06	2.80
1.42	13.11	0.57	20.26	1.07	30.82	5.22	31.05	3.29	32.05	5.86	31.96	0.80	37.74	1.23
1.67	12.70	0.32	20.50	2.65	32.42	5.47	34.57	3.56	35.06	5.60	30.13	4.89	34.64	3.61
1.92	13.32	0.75	24.52	1.28	34.61	3.39	36.31	4.09	37.68	3.35	33.64	7.16	35.83	3.03
2.17	13.83	1.62	24.67	3.88	38.83	5.34	33.33	5.18	29.69	2.92	36.79	6.40	36.74	8.28
2.42	14.40	1.49	24.59	0.96	37.53	5.85	34.55	5.82	32.02	4.13	28.93	1.95	33.99	6.15
2.67	14.41	3.16	24.13	1.96	38.64	5.71	35.28	7.26	33.49	3.72	29.27	5.32	31.75	4.51
2.92	14.69	3.37	23.12	4.31	39.51	3.32	40.57	10.95	31.01	2.21	26.96	2.74	30.03	3.17
3.17	13.09	1.62	22.45	2.46	33.71	4.22	40.47	13.30	33.68	5.83	25.49	2.83	35.48	5.36
3.42	13.13	2.61	21.93	3.17	36.78	6.20	37.97	8.55	34.42	6.82	30.84	3.63	29.41	2.26
3.67	13.79	2.57	22.14	3.83	38.68	6.83	37.04	10.94	36.29	9.33	25.99	0.74	31.78	3.42
3.92	11.99	1.88	20.92	1.40	35.01	5.00	41.15	13.68	37.18	10.76	27.10	4.22	32.48	6.88
4.17	11.36	1.85	22.06	3.34	41.09	13.84	51.44	19.08	32.03	4.85	32.51	6.11	34.93	7.88
4.42	13.27	2.69	25.82	7.39	42.06	20.66	57.01	25.31	33.81	10.03	27.65	2.23	31.03	5.40
4.67	13.56	3.03	24.39	3.68	52.46	25.31	54.30	21.13	34.96	10.04	32.87	8.73	30.64	4.00
4.92	13.70	1.19	26.81	4.90	48.84	16.63	64.30	27.01	35.76	9.39	29.00	8.83	32.54	6.01
5.17	12.21	1.97	30.79	8.02	43.24	13.18	55.36	22.26	39.20	10.84	27.94	6.07	29.69	6.47
5.42	11.35	0.98	27.25	5.92	77.16	48.50	49.93	16.90	38.36	8.32	36.99	9.18	30.43	3.58
5.67	13.22	3.89	26.91	5.48	66.34	26.44	67.47	28.67	43.82	12.16	29.38	6.10	32.08	6.42
5.92	12.59	2.62	33.06	10.06	101.62	71.97	81.41	42.02	36.41	9.00	31.36	8.83	29.54	5.66
6.17	12.54	3.62	38.66	13.00	94.27	45.30	106.11	72.46	40.52	14.09	37.97	17.59	27.69	4.80
6.42	12.40	2.59	39.38	10.26	95.47	46.59	68.51	26.54	44.60	9.53	28.07	5.97	31.22	7.05
6.67	11.98	1.90	36.92	9.15	93.07	41.88	88.18	57.58	39.02	10.98	27.45	5.16	35.82	11.32
6.92	12.63	1.25	40.04	10.68	135.60	78.41	208.77	140.46	44.68	14.99	31.08	6.76	35.12	9.99

Table S5 Kinetic K_D data for the formation of the ternary 14-3-3/Pin1/FC-A complex. Average K_D values (av.), and corresponding standard deviations (s.d.) are given in µM.

Incub. Time (h)	**FC-A** stabilized 14-3-3/Pin1 complex													
	**pH 6.0**		**pH 6.5**		**pH 7.0**		**pH 7.5**		**pH 8.0**		**pH 8.5**		**pH 9.0**	
	av.	s.d.	av.	s.d.	av.	s.d.	av.	s.d.	av.	s.d.	av.	s.d.	av.	s.d.
0.17	3.04	0.24	2.89	0.27	3.13	0.46	2.91	0.21	3.45	0.28	3.00	0.19	3.13	0.45
0.42	3.27	0.27	2.95	0.25	3.27	0.42	3.03	0.19	3.59	0.30	3.18	0.16	3.35	0.46
0.67	3.43	0.35	3.16	0.36	3.31	0.50	3.13	0.14	3.72	0.41	3.27	0.28	3.43	0.57
0.92	3.43	0.35	3.16	0.36	3.31	0.50	3.13	0.14	3.72	0.41	3.27	0.28	3.43	0.57
1.17	3.41	0.37	3.18	0.31	3.46	0.51	3.16	0.14	3.76	0.37	3.38	0.27	3.42	0.50
1.42	3.47	0.32	3.23	0.38	3.49	0.52	3.15	0.11	3.88	0.47	3.37	0.25	3.46	0.57
1.67	3.56	0.39	3.30	0.37	3.51	0.49	3.23	0.15	3.96	0.34	3.32	0.24	3.51	0.57
1.92	3.54	0.44	3.26	0.39	3.56	0.57	3.32	0.24	3.90	0.35	3.36	0.27	3.53	0.52
2.17	3.68	0.39	3.22	0.36	3.56	0.58	3.26	0.28	3.99	0.46	3.35	0.19	3.50	0.46
2.42	3.62	0.37	3.24	0.33	3.64	0.56	3.27	0.15	3.95	0.49	3.39	0.28	3.48	0.43
2.67	3.67	0.29	3.37	0.35	3.63	0.51	3.32	0.20	3.98	0.40	3.47	0.24	3.62	0.44
2.92	3.78	0.39	3.37	0.33	3.78	0.51	3.39	0.15	3.94	0.33	3.50	0.26	3.51	0.43
3.17	3.71	0.39	3.40	0.33	3.67	0.56	3.39	0.20	3.91	0.34	3.44	0.26	3.53	0.47
3.42	3.82	0.46	3.39	0.34	3.76	0.49	3.39	0.13	3.95	0.34	3.51	0.30	3.60	0.43
3.67	3.85	0.45	3.41	0.32	3.82	0.41	3.54	0.22	4.08	0.28	3.53	0.16	3.58	0.45
3.92	3.81	0.28	3.31	0.30	3.92	0.63	3.51	0.16	4.04	0.41	3.60	0.28	3.57	0.50
4.17	3.83	0.37	3.51	0.30	3.93	0.53	3.67	0.22	4.05	0.35	3.61	0.25	3.64	0.45
4.42	3.82	0.50	3.43	0.32	3.92	0.53	3.62	0.21	4.09	0.37	3.62	0.30	3.72	0.50
4.67	3.84	0.41	3.43	0.38	3.92	0.52	3.65	0.29	4.15	0.42	3.57	0.27	3.65	0.44
4.92	3.75	0.43	3.48	0.36	4.03	0.55	3.61	0.24	4.25	0.28	3.62	0.22	3.62	0.43
5.17	3.81	0.35	3.53	0.37	4.09	0.57	3.70	0.24	4.31	0.39	3.61	0.29	3.70	0.54
5.42	3.81	0.35	3.56	0.37	4.13	0.57	3.78	0.25	4.24	0.34	3.57	0.14	3.68	0.48
5.67	3.78	0.36	3.55	0.36	4.14	0.59	3.74	0.28	4.21	0.40	3.68	0.29	3.85	0.47
5.92	3.82	0.34	3.56	0.40	4.18	0.54	3.82	0.32	4.22	0.38	3.59	0.22	3.73	0.52
6.17	3.83	0.28	3.54	0.33	4.28	0.58	3.89	0.26	4.32	0.44	3.79	0.28	3.81	0.50
6.42	3.84	0.40	3.55	0.33	4.34	0.59	3.94	0.28	4.29	0.38	3.63	0.24	3.73	0.54
6.67	3.83	0.37	3.61	0.38	4.36	0.71	3.87	0.25	4.28	0.38	3.75	0.23	3.78	0.60
6.92	3.84	0.41	3.62	0.37	4.38	0.62	4.01	0.27	4.19	0.36	3.72	0.23	3.77	0.52

Table S6 Kinetic K_D data for the formation of the ternary 14-3-3/Pin1/MG1 complex. Average K_D values (av.), and corresponding standard deviations (s.d.) are given in µM.

Incub. Time (h)	**MG1** stabilized 14-3-3/Pin1 complex													
	**pH 6.0**		**pH 6.5**		**pH 7.0**		**pH 7.5**		**pH 8.0**		**pH 8.5**		**pH 9.0**	
	av.	s.d.	av.	s.d.	av.	s.d.	av.	s.d.	av.	s.d.	av.	s.d.	av.	s.d.
0.17	5.10	0.50	5.22	0.29	5.20	0.16	5.37	1.45	3.16	0.40	2.42	0.18	2.86	0.27
0.42	6.28	0.77	5.51	0.27	3.79	0.24	3.20	0.90	1.52	0.25	1.03	0.13	1.19	0.09
0.67	6.06	0.44	5.51	0.27	3.16	0.44	2.21	0.77	0.96	0.20	0.60	0.07	0.71	0.06
0.92	6.06	0.44	5.51	0.27	3.16	0.44	2.21	0.77	0.96	0.20	0.60	0.07	0.71	0.06
1.17	5.87	0.47	5.42	0.38	3.21	0.40	2.00	0.64	0.85	0.18	0.56	0.07	0.64	0.07
1.42	6.03	0.72	5.48	0.34	3.03	0.29	1.98	0.72	0.82	0.19	0.50	0.06	0.59	0.05
1.67	6.02	0.35	5.47	0.45	2.92	0.26	1.78	0.65	0.75	0.18	0.48	0.05	0.55	0.04
1.92	5.98	0.42	5.23	0.36	3.00	0.35	1.73	0.61	0.75	0.15	0.46	0.06	0.55	0.05
2.17	5.96	0.75	5.23	0.28	2.87	0.26	1.67	0.57	0.71	0.15	0.45	0.04	0.50	0.04
2.42	5.76	0.48	5.44	0.23	2.88	0.34	1.65	0.56	0.69	0.14	0.43	0.03	0.51	0.06
2.67	5.48	0.39	5.61	0.40	2.80	0.31	1.70	0.55	0.67	0.13	0.41	0.04	0.50	0.05
2.92	5.33	0.49	5.17	0.35	2.81	0.17	1.60	0.54	0.67	0.14	0.42	0.05	0.47	0.02
3.17	5.52	0.56	5.28	0.46	2.89	0.35	1.59	0.57	0.66	0.11	0.42	0.04	0.49	0.04
3.42	5.39	0.50	5.46	0.24	2.91	0.39	1.58	0.56	0.65	0.15	0.40	0.05	0.46	0.04
3.67	5.41	0.31	5.27	0.29	2.89	0.26	1.56	0.54	0.64	0.14	0.41	0.06	0.46	0.02
3.92	5.26	0.30	5.03	0.25	2.81	0.29	1.53	0.49	0.64	0.14	0.40	0.05	0.46	0.04
4.17	5.20	0.20	5.34	0.34	2.84	0.42	1.51	0.49	0.65	0.12	0.40	0.05	0.46	0.04
4.42	5.05	0.45	5.47	0.65	2.87	0.35	1.58	0.52	0.63	0.14	0.39	0.05	0.46	0.03
4.67	5.36	0.02	5.50	0.46	2.96	0.30	1.58	0.54	0.62	0.13	0.39	0.05	0.45	0.04
4.92	4.97	0.34	5.49	0.62	2.76	0.28	1.50	0.48	0.62	0.11	0.38	0.05	0.46	0.04
5.17	5.32	0.41	5.46	0.34	2.73	0.31	1.56	0.48	0.61	0.12	0.39	0.07	0.44	0.01
5.42	4.98	0.47	5.28	0.39	2.80	0.42	1.51	0.48	0.62	0.12	0.39	0.04	0.45	0.03
5.67	4.88	0.52	5.45	0.56	2.87	0.37	1.57	0.53	0.59	0.11	0.39	0.04	0.45	0.03
5.92	5.17	0.52	5.44	0.68	2.85	0.40	1.56	0.54	0.61	0.14	0.38	0.04	0.44	0.02
6.17	4.86	0.66	5.34	0.66	2.62	0.23	1.50	0.51	0.59	0.12	0.38	0.05	0.45	0.02
6.42	4.57	0.58	5.29	0.41	2.72	0.36	1.53	0.57	0.59	0.11	0.38	0.05	0.44	0.02
6.67	4.67	0.21	5.61	0.72	2.76	0.25	1.47	0.47	0.60	0.11	0.38	0.05	0.45	0.04
6.92	4.44	0.58	5.26	0.64	2.88	0.38	1.55	0.46	0.57	0.10	0.37	0.05	0.44	0.02

Table S7 Kinetic K_D data for the formation of the binary 14-3-3/Pin1 complex and the MG2-6 stabilized 14-3-3/Pin1 complex. Average K_D values (av.), and corresponding standard deviations (s.d.) are given in µM.

Incub. Time (h)	Binary 14-3-3/Pin1 complex								**MG2** stabilized 14-3-3/Pin1 complex							
	**pH 6.5**		**pH 7.0**		**pH 7.5**		**pH 8.0**		**pH 6.5**		**pH 7.0**		**pH 7.5**		**pH 8.0**	
	av.	s.d.	av.	s.d.	av.	s.d.	av.	s.d.	av.	s.d.	av.	s.d.	av.	s.d.	av.	s.d.
0.17	19.72	1.45	29.99	3.55	34.99	5.12	32.83	8.08	16.71	1.68	23.12	3.63	24.42	3.88	23.59	2.82
0.42	19.96	1.53	29.88	3.67	32.88	5.14	34.55	1.93	18.11	1.63	22.79	2.18	22.47	3.44	19.23	1.37
0.67	19.78	1.67	27.73	1.98	33.14	5.98	33.83	5.00	17.88	1.95	20.52	0.80	22.51	3.03	18.95	1.53
0.92	21.75	1.33	27.04	2.46	34.87	5.52	34.22	4.24	17.31	1.08	22.61	0.93	22.33	2.94	17.89	2.14
1.17	18.44	1.86	29.27	5.89	33.78	3.61	35.58	6.05	18.06	1.09	22.12	2.70	21.34	2.78	16.00	1.47
1.42	21.26	1.33	28.85	2.08	35.12	4.04	39.05	9.44	17.90	1.93	22.45	0.57	21.36	3.03	16.25	2.20
1.67	21.73	3.36	29.93	2.80	32.39	2.97	35.60	2.86	17.92	2.76	22.18	2.26	22.51	4.20	17.00	2.92
1.92	21.87	2.69	29.32	2.53	33.55	4.59	41.07	10.22	18.06	0.94	23.53	4.75	21.42	1.93	15.80	1.29
2.17	21.23	3.08	32.25	4.65	36.64	8.15	39.57	9.21	18.29	1.58	24.22	4.45	22.83	3.94	16.80	2.38
2.42	22.40	4.41	34.66	3.19	39.72	1.37	41.00	5.49	18.29	0.65	22.08	4.50	22.51	2.63	16.57	1.96
2.67	23.33	2.00	39.71	9.74	40.34	8.98	38.33	4.51	19.52	3.20	26.22	4.61	22.71	2.40	15.75	1.04
2.92	24.18	2.71	34.88	3.75	43.12	7.41	39.33	5.68	18.88	2.09	22.68	2.49	21.42	2.27	16.90	2.83
3.17	22.76	2.56	36.19	3.45	45.08	7.63	41.12	2.25	19.62	5.27	22.76	1.23	21.00	3.25	17.09	2.13
3.42	27.18	3.55	47.98	13.10	44.53	12.31	37.34	3.65	18.89	0.69	24.63	2.81	26.04	5.05	18.23	2.36
3.67	24.72	3.34	39.25	6.36	44.60	6.36	36.60	5.29	20.17	3.57	26.47	2.65	23.36	5.35	15.76	1.70
3.92	25.10	0.97	40.24	2.91	42.62	8.16	42.17	1.89	20.16	3.81	24.60	4.96	22.55	2.57	15.83	2.78

Incub. Time (h)	**MG3** stabilized 14-3-3/Pin1 complex								**MG4** stabilized 14-3-3/Pin1 complex							
	**pH 6.5**		**pH 7.0**		**pH 7.5**		**pH 8.0**		**pH 6.5**		**pH 7.0**		**pH 7.5**		**pH 8.0**	
	av.	s.d.	av.	s.d.	av.	s.d.	av.	s.d.	av.	s.d.	av.	s.d.	av.	s.d.	av.	s.d.
0.17	9.53	0.27	12.81	0.22	13.94	0.82	12.89	1.76	7.20	0.98	7.32	0.74	7.07	1.36	6.49	0.58
0.42	8.93	0.69	9.01	0.37	7.89	0.88	6.79	1.35	6.67	1.17	5.14	0.82	3.61	1.03	2.76	0.42
0.67	8.38	0.42	7.70	0.74	5.28	0.55	4.42	0.55	6.36	1.40	4.18	0.70	2.63	0.87	1.90	0.29
0.92	8.90	0.68	6.69	0.45	4.39	0.45	3.52	0.53	6.40	1.59	3.94	0.89	2.24	0.78	1.66	0.28
1.17	7.91	0.69	6.48	0.80	3.76	0.43	3.01	0.54	6.53	1.61	3.53	0.78	1.98	0.72	1.53	0.28
1.42	7.88	0.44	6.05	0.36	3.46	0.30	2.58	0.60	6.59	1.58	3.60	0.78	1.90	0.67	1.39	0.20
1.67	7.84	0.63	5.90	0.56	3.30	0.30	2.38	0.65	6.27	1.14	3.40	0.77	1.73	0.61	1.26	0.20
1.92	7.59	0.68	5.69	0.72	3.00	0.37	2.17	0.53	6.48	1.30	3.31	0.65	1.72	0.67	1.19	0.15
2.17	7.71	0.68	5.48	0.66	2.94	0.30	2.05	0.58	6.30	1.46	3.37	0.75	1.66	0.58	1.20	0.14
2.42	7.68	0.57	5.45	0.60	2.81	0.32	1.93	0.68	6.26	1.50	3.32	0.66	1.63	0.59	1.15	0.20
2.67	7.50	0.68	5.61	0.75	2.77	0.41	1.88	0.64	5.93	1.21	3.37	0.67	1.57	0.59	1.15	0.17
2.92	7.56	0.36	5.26	0.35	2.74	0.35	1.82	0.58	5.83	1.22	3.38	0.64	1.63	0.58	1.10	0.16
3.17	7.35	0.60	5.50	0.58	2.61	0.26	1.76	0.63	6.20	1.39	3.32	0.62	1.56	0.52	1.10	0.14
3.42	7.92	0.62	5.49	0.68	2.56	0.29	1.68	0.63	6.08	1.33	3.26	0.59	1.57	0.54	1.07	0.15
3.67	7.53	0.56	5.18	0.73	2.37	0.16	1.65	0.65	6.19	1.60	3.31	0.65	1.50	0.57	1.07	0.17
3.92	7.25	0.78	5.08	0.47	2.42	0.32	1.57	0.59	5.78	1.25	3.47	0.78	1.58	0.56	1.04	0.15

Incub. Time (h)	**MG5** stabilized 14-3-3/Pin1 complex								**MG6** stabilized 14-3-3/Pin1 complex							
	**pH 6.5**		**pH 7.0**		**pH 7.5**		**pH 8.0**		**pH 6.5**		**pH 7.0**		**pH 7.5**		**pH 8.0**	
	av.	s.d.	av.	s.d.	av.	s.d.	av.	s.d.	av.	s.d.	av.	s.d.	av.	s.d.	av.	s.d.
0.17	7.55	0.42	9.68	0.53	8.83	0.53	7.74	1.51	11.66	0.49	15.83	1.16	16.20	2.22	15.99	1.89
0.42	7.89	1.17	7.49	0.54	4.99	0.40	2.96	0.55	11.11	0.88	12.10	0.96	11.24	1.90	9.56	0.43
0.67	8.01	1.29	6.56	0.50	3.68	0.59	2.14	0.49	10.48	0.88	9.54	0.36	8.01	0.97	6.22	0.21
0.92	7.51	0.71	7.12	1.49	3.15	0.54	1.80	0.43	10.22	0.63	8.68	1.38	6.71	0.76	4.38	0.29
1.17	8.60	1.18	6.00	0.59	3.02	0.48	1.56	0.38	10.06	1.30	8.27	1.19	5.82	0.62	3.82	0.33
1.42	7.89	1.32	6.10	0.43	2.81	0.43	1.43	0.38	10.05	1.07	7.51	0.96	5.23	0.55	3.24	0.34
1.67	7.79	1.08	5.85	0.67	2.61	0.50	1.36	0.32	10.04	1.02	6.85	0.75	4.73	0.61	2.94	0.37
1.92	7.71	1.22	5.92	0.65	2.59	0.61	1.33	0.36	9.23	1.18	6.78	0.85	4.45	0.35	2.71	0.43
2.17	7.95	1.09	5.78	0.71	2.56	0.48	1.25	0.33	9.89	1.10	7.05	0.87	4.27	0.35	2.50	0.35
2.42	7.49	1.02	5.89	0.65	2.62	0.53	1.26	0.34	9.21	0.91	6.76	1.08	4.08	0.41	2.38	0.32
2.67	7.89	1.26	5.78	0.59	2.44	0.50	1.20	0.30	9.77	0.78	6.50	0.76	4.03	0.27	2.22	0.31
2.92	7.92	0.85	5.72	0.28	2.54	0.45	1.24	0.32	9.46	0.67	6.55	0.88	3.88	0.41	2.19	0.33
3.17	7.65	1.26	6.02	0.71	2.50	0.53	1.14	0.35	9.44	0.91	6.38	1.01	3.75	0.30	2.13	0.26
3.42	7.91	1.17	5.67	0.53	2.47	0.58	1.16	0.38	9.88	1.29	6.92	0.86	3.65	0.38	2.07	0.34
3.67	7.98	1.10	5.93	0.55	2.41	0.43	1.18	0.34	9.89	0.97	6.68	1.10	3.60	0.24	1.95	0.30
3.92	7.79	1.50	5.75	0.25	2.28	0.50	1.16	0.34	9.51	0.62	6.29	0.80	3.51	0.36	1.93	0.34

Table S8 Kinetic K_D data for the formation of the binary 14-3-3/p65 complex. Average K_D values (av.), and corresponding standard deviations (s.d.) are given in µM.

Incub. Time (h)	Binary 14-3-3/p65 complex													
	pH 6.0		pH 6.5		pH 7.0		pH 7.5		pH 8.0		pH 8.5		pH 9.0	
	av.	s.d.	av.	s.d.	av.	s.d.	av.	s.d.	av.	s.d.	av.	s.d.	av.	s.d.
0.17	115.35	2.09	121.13	2.49	146.00	4.15	162.91	3.95	161.88	6.48	162.94	2.51	178.81	17.70
0.42	138.42	1.53	143.72	6.04	162.23	4.57	180.20	11.87	183.57	9.98	189.01	1.20	198.78	12.19
0.67	139.53	3.92	157.47	11.77	168.50	13.82	183.52	4.12	185.46	6.28	191.54	4.64	179.03	28.34
0.92	139.53	3.92	150.39	3.47	164.41	9.14	182.63	3.13	183.52	3.60	194.13	2.93	194.08	10.98
1.17	144.37	0.90	144.08	3.04	162.67	5.28	182.32	7.38	185.24	1.88	196.30	6.56	190.63	8.67
1.42	139.31	2.69	143.82	4.78	161.30	8.35	179.29	1.40	187.44	3.95	193.37	2.77	193.66	10.69
1.67	139.46	2.02	146.08	3.49	164.09	9.02	180.17	1.29	182.29	4.55	194.29	4.27	192.96	10.44
1.92	141.32	4.15	141.45	2.47	158.17	5.16	177.15	3.01	178.01	7.08	190.31	6.42	188.23	11.28
2.17	141.35	2.84	145.10	4.26	161.92	9.93	173.21	5.70	175.57	4.36	193.42	2.12	187.67	7.98
2.42	140.66	1.91	141.31	3.51	157.82	7.18	174.59	2.74	174.65	4.97	184.47	3.01	187.68	7.28
2.67	142.20	1.88	150.59	12.64	161.08	8.15	175.34	8.37	171.25	6.38	184.43	6.83	172.49	23.65
2.92	144.51	2.81	147.35	13.40	161.14	3.82	172.44	4.08	172.85	8.07	182.33	6.02	176.04	26.37
3.17	142.55	3.29	147.12	10.91	158.88	7.70	172.65	5.44	172.08	4.98	180.26	11.48	170.78	24.11
3.42	141.80	3.80	150.86	13.29	159.17	2.75	169.83	5.75	172.84	2.36	184.61	5.13	175.39	27.70
3.67	138.60	5.38	149.01	11.16	155.67	8.60	166.39	2.17	170.10	7.51	174.12	7.37	175.83	25.71
3.92	144.57	2.37	144.27	11.24	158.93	4.29	170.05	1.95	167.74	3.62	180.18	5.64	173.55	28.98

Table S9 Kinetic K_D data for the formation of the MG7 stabilized 14-3-3/p65 complex. Average K_D values (av.), and corresponding standard deviations (s.d.) are given in µM.

Incub. Time (h)	**MG7** stabilized 14-3-3/p65 complex													
	pH 6.0		pH 6.5		pH 7.0		pH 7.5		pH 8.0		pH 8.5		pH 9.0	
	av.	s.d.	av.	s.d.	av.	s.d.	av.	s.d.	av.	s.d.	av.	s.d.	av.	s.d.
0.17	54.64	10.93	54.24	4.50	66.48	11.78	60.59	10.29	46.24	5.21	41.80	4.35	38.13	5.07
0.42	62.99	11.31	60.49	9.61	51.20	9.21	40.41	4.05	24.93	2.83	20.49	1.13	19.23	3.74
0.67	65.05	9.93	58.60	5.51	43.92	3.29	28.02	3.86	16.16	1.94	12.10	1.68	10.82	1.79
0.92	65.05	9.93	58.60	5.51	43.92	3.29	28.02	3.86	16.16	1.94	12.10	1.68	10.82	1.79
1.17	67.27	6.32	63.68	5.75	44.02	4.01	25.20	2.43	15.05	0.76	11.42	1.81	9.92	1.65
1.42	68.78	10.98	57.75	8.95	38.61	3.19	23.64	2.92	14.27	0.37	10.62	1.19	9.05	1.34
1.67	71.08	7.59	64.01	5.75	38.65	2.46	23.86	2.51	14.33	0.99	10.38	1.14	8.62	1.57
1.92	70.76	11.65	61.61	7.99	39.06	4.18	23.25	1.68	12.92	1.10	10.10	1.07	8.34	1.26
2.17	81.07	16.30	60.47	7.53	37.99	1.65	22.17	2.40	11.92	0.19	9.96	1.48	8.43	1.65
2.42	71.95	5.23	62.45	3.26	34.45	1.98	20.93	1.96	11.89	0.13	9.12	1.32	8.43	1.35
2.67	60.38	6.73	60.72	8.97	38.11	6.81	23.80	2.06	11.92	0.17	10.04	1.46	7.87	1.63
2.92	63.93	11.51	58.76	4.86	37.26	3.05	21.83	1.90	11.43	0.25	9.76	1.54	7.81	1.42
3.17	64.92	14.80	55.98	5.02	35.79	1.16	21.42	1.93	11.74	0.73	9.48	1.39	8.01	1.33
3.42	76.28	24.49	56.16	5.20	37.22	4.55	20.69	1.52	11.54	0.77	9.39	0.99	7.96	1.13
3.67	61.51	2.76	56.66	5.51	37.11	6.49	20.93	3.06	11.24	0.54	9.26	0.69	8.31	1.30
3.92	63.07	10.22	55.15	9.73	37.36	3.54	22.37	2.58	12.46	1.21	9.00	0.49	8.02	1.21

5. Experimental Section – Chemistry

Table S10 Compound overview.

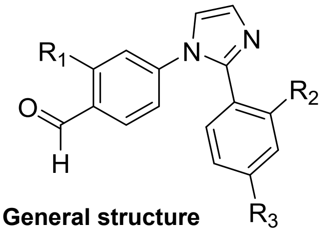

General structure 14-3-3/Pin1 stabilizers

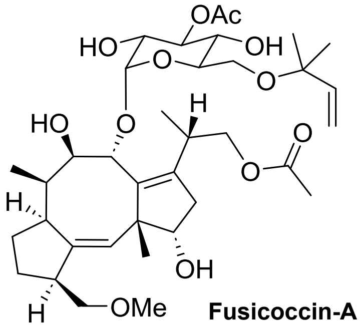

Fusicoccin-A

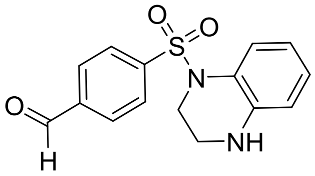

A24j

Manuscript name	*Chemical name*	*R_1*	*R_2*	*R_3*	*Preparation*
**MG1**	2-bromo-4-(2-(2,4-difluorophenyl)-1*H*-imidazol-1-yl)benzaldehyde	Br	F	F	*Cossar et al.*[1]
**MG2**	2-methyl-4-(2-phenyl-1*H*-imidazol-1-yl)benzaldehyde	CH_3	H	H	*Cossar et al.*[1]
**MG3**	2-iodo-4-(2-phenyl-1*H*-imidazol-1-yl)benzaldehyde	I	H	H	*This manuscript*
**MG4**	2-bromo-4-(2-phenyl-1*H*-imidazol-1-yl)benzaldehyde	Br	H	H	*Cossar et al.*[1]
**MG5**	2-chloro-4-(2-phenyl-1*H*-imidazol-1-yl)benzaldehyde	Cl	H	H	*Cossar et al.*[1]
**MG6**	4-(2-phenyl-1*H*-imidazol-1-yl)-2-(trifluoromethyl)benzaldehyde	CF_3	H	H	*Cossar et al.*[1]
**Fusicoccin-A (FC-A)**	(*S*)-2-((1*S*,4*R*,5*R*,6*R*,6a*S*,9*S*,10a*R*,*E*)-4-(((2*S*,3*R*, 4*S*,5*R*,6*R*)-4-acetoxy-3,5-dihydroxy-6-(((2-methyl but-3-en-2-yl)oxy)methyl)tetrahydro-2*H*-pyran-2-yl)oxy)-1,5-dihydroxy-9-(methoxymethyl)-6,10a-dimethyl-1,2,4,5,6,6a,7,8,9,10adecahydrodicyclo penta[*a*,*d*][8]annulen-3-yl)propyl acetate				*Cossar et al.*[1]
**MG7**	4-((3,4-dihydroquinoxalin-1(2*H*)-yl)sulfonyl)benzaldehyde				*Wolter et al.*[3]

General materials

All reactions were prepared using analytical grade (AR) grade solvents. All reagents were purchased from TCI, or Sigma-Aldrich and were used without further purification. Solvents were removed in vacuo using a Buchi rotary evaporator connected to a diaphragm pump. All other used solvents were of analytical grade and supplied by Biosolve. Reaction glassware was dried at 130 °C for more than 24 hours prior to use. TLC was carried out on aluminum-backed silica (Merck silica gel 60 F254) plates supplied by Merck. Visualization of the plates was achieved using an ultraviolet lamp (λ_{max} = 254 nm). Preparative HPLC was performed using a Gemini S4 110A 150 x 21.20 mm column using miliQ water with 0.1% formic acid (FA) and acetonitrile (ACN) with 0.1% FA. Analytical (LR) HPLC-MS analysis was performed on a system using comprising a C4 Jupiter SuC4300A 150 x 2.0 mm column using miliQ water with 0.1% FA and acetonitrile with 0.1% FA, using a gradient of 5% to 100% ACN over 10 minutes, connected to a Thermo Fisher LTQ XL Linear Ion Trap Mass Spectrometer. The purity of the samples was assessed using PDA (254 nm) and MS (positive mode, m/z 100 – 1000). Unless otherwise stated all final compounds were ≥95% pure as judged by HPLC. High resolution mass spectra (HRMS) were recorded using a Waters ACQUITY UPLC I-Class LC system coupled to a Xevo G2 Quadrupole Time of Flight (Q-TOF) mass spectrometer equipped with a Phenomex kinetex® 2.6 µm EVO C18 100 x 2.1 mm column. Proton (^{1}H) and carbon (^{13}C) NMR spectral data were collected on a 400 MHz Bruker Cryomagnet. Chemical shifts (δ) are quoted in parts per million (ppm) and referenced to the residual solvent peak. Coupling constants (J) are quoted in Hertz (Hz) and splitting patterns reported in an abbreviated manner: app. (apparent), s (singlet), d (doublet), t (triplet), q (quartet), and m (multiplet). Assignments were made with the aid of 2D COSY, HMQC, and HMBC experiments.

Synthetic procedures and Characterization

2-iodo-4-(2-phenyl-1*H*-imidazol-1-yl)benzaldehyde (**MG3**)

A round bottom flask equipped with a reflux condenser was charged with 119.9 mg 2-bromo-4-(2-phenyl-1*H*-imidazol-1-yl)benzaldehyde (0.366 mmole, **MG4**), 3.5 mg copper(I) iodide (0.0183 mmole), 109.9 mg sodium iodide (0.733 mmole), and 3.2 mg *N,N'*-dimethylethylenediamine (0.0366 mmole) in 8 mL dioxane under argon (protocol adapted from Klapars *et al.*[18]). The reaction mixture was stirred at 110 °C for 72 hours. The resulting suspension was allowed to reach room temperature, diluted with 25% ammonia (25 mL), diluted with water (100 mL), and extracted with ethyl acetate (3 x 50 mL). The organic layers were combined, and washed with a 1% ammonia solution (2 x 100 mL), a citric acid solution (pH 4, 3 x 50 mL), and brine (50 mL). The organic layer was dried over magnesium sulfate, and concentrated in vacuo. The residue was purified by preparative HPLC (isocratic, 25% ACN in miliQ water (+ 0.1% FA)) to afford a white powder (26.5 mg, 19% yield). LRMS (ESI+) *m/z* 375 (M+H); HRMS (ESI+) calculated for $C_{16}H_{12}IN_2O$ (M+H), *m/z* 374.9994; found *m/z* 374.9995; ^{1}H NMR (399 MHz, Acetone-d6) δ 10.05 (s, 1H), 8.01 (s, 1H), 7.87 (d, J = 8.2 Hz, 1H), 7.52 (s, 1H), 7.47 (d, J = 2.0 Hz, 1H), 7.43 – 7.32 (m, 5H), 7.22 (s, 1H); ^{13}C NMR (100 MHz, Acetone-d6) δ 194.7, 147.3, 144.6, 138.2, 135.4, 131.5, 131.4, 130.5, 129.7, 129.5, 129.2, 127.1, 123.6, 100.4.

6. Experimental Section – Native mass spectrometry

Analytical grade ammonium acetate and LC-MS grade Dimethyl Sulfoxide (DMSO) were purchased from Fisher Scientific. 14-3-3γ was buffer exchanged into 100 mM ammonium acetate (pH 8.0) using a 30 kDa molecular weight cut-off Amicon Ultra centrifugal filter (Merck Millipore) and stored at -80 °C prior to use. The unlabeled Pin1 17mer peptide (Ac-LVKHSQSRRPSpSWRQEK, Genscript) was diluted into 100mM ammonium acetate at pH 6.5, 7.5 and 8.0 dependent on the experiment performed. To form the 14-3-3/Pin1 complex, 14-3-3γ (10 μM) was mixed with the Pin1 17mer peptide (50 μM). To form the stabilized 14-3-3/Pin1 complex, 14-3-3γ (10 μM) was incubated with both a 5-fold excess of the Pin1 17mer peptide (50 μM) and **MG1** (50 μM) in 100 mM ammonium acetate pH 6.5, pH 7.5 or pH 8.0 and the samples incubated for various time periods at 22 ºC. At 15 minutes, 1 hour, 2 hours, 4 hours and 20 hours aliquots were taken, immediately diluted 1:5 into 100 mM ammonium acetate pH 6.8 and directly infused into the mass spectrometer. A final concentration of 1.25 % DMSO was used for all experiments.

All native MS experiments were performed on an Orbitrap Eclipse Tribrid mass spectrometer (Thermo Fisher Scientific, Bremen) coupled to a nanoelectrospray source that used gold-coated borosilicate glass capillaries, pulled in-house. Positive ionization mode was used throughout with the capillary voltage set to 1.2 kV. The source temperature was set at 275 °C, in-source dissociation at 25, S-lens RF at 100. High pressure mode was used and a mass range of 2000-8000 *m/z* used to monitor the binding equilibria. Mass spectra were acquired using a maximum ion injection time of 50 ms. The automatic gain control was set to $1x10^6$ and the ions detected in the Orbitrap with resolution set to 7,500. To check **MG1** did not bind to Pin1 alone, a mass range of 100-4000 *m/z* was used with in-source dissociation set to 0. To detect covalent modification of 14-3-3γ with **MG1**, all peaks within 4000-5500 *m/z* range were selected in the ion trap and subjected to higher-energy collision induced dissociation (HCD) using nitrogen as the collision gas and an absolute HCD energy set to 140. MS/MS data were acquired over a 500-8000 *m/z* range with the product ions detected in the Orbitrap with a resolution set to 7,500. All data was analyzed using XCalibur (*v*.4.1). All proteins and protein complexes were identified based on their close matches to their theoretical mass (Table S11). In the cases where peak overlap was observed (for example with $(14\text{-}3\text{-}3_2)^{12+}$ and $(14\text{-}3\text{-}3/\text{PIN1}_2/\textbf{MG1}_2)^{13+}$ ions) adjacent charge states for the protein complex of question were sought and compared with the MS/MS data to confirm their identity. In cases whereby complex identity was still ambiguous and complex overlap was envisaged (for example with 1 hour, 2 hour and 4 hour time-points), these charge states were excluded from all calculations.

To quantify each complex, the relative abundance of the complexes observed was assumed to reflect their abundance in solution. For simplicity, the quantified complexes included in all calculations were: 14-3-3_2, 14-3-3_2/Pin1, 14-3-3_2/PIN1_2, 14-3-3_2/Pin1/**MG1**, 14-3-3_2/PIN1_2/**MG1** and 14-3-3_2/PIN1_2/**MG1**$_2$ whereby the relative abundance of each complex was calculated as a percentage of the sum of all other complexes. To minimize issues with overlapping peaks distorting the quantification data, only the top two charge states for each complex were included in the calculations. The charge states chosen for each complex are highlighted in Table S11. To determine the extent of covalent stabilization, the 14-3-3/**MG1** complex from within the MS/MS data was plotted as a percentage of the total 14-3-3 protein signal observed (i.e. 14-3-3 and 14-3-3/**MG1** signals combined).

Table S11 Theoretical and measured masses of protein complexes. * includes -18 Da or -36 Da for one or two **MG1** covalently bound to 14-3-3, respectively. The error reported is the standard deviation measured between charge states. The two most abundant charge states are noted for all complexes included in quantitative measurements. All masses reported were from the 1 h timepoint data.

Protein complex	Theoretical mass (Da)	Measured mass (Da)	Charge states included for quantification
14-3-3	31543.0	31542.8± 0.1	N/A
Pin1 peptide	2231.1	2231.1±0.1	N/A
14-3-3/Pin1	33774.1	33773.7 ± 0.5	N/A
14-3-3/Pin1/**MG1**	34119.3*	34119.7 ± 1.3	N/A
14-3-3/**MG1**	31888.2*	31888.8 ± 0.4	N/A
14-3-3_2	63086.0	63090.7 ± 1.0	14+, 13+
14-3-3_2/Pin1	65317.1	65321.9 ± 0.6	14+, 13+
14-3-3_2/Pin1_2	67548.2	67553.6 ± 0.1	14+, 13+
14-3-3_2/**MG1**	63431.2*	63431.3 ± 1.6	N/A
14-3-3_2/**MG1**$_2$	63776.3*	63778.8 ± 2.3	N/A
14-3-3_2/Pin1/**MG1**	65662.3*	65666.9 ± 1.1	14+, 13+
14-3-3_2/Pin1_2/**MG1**	67893.4*	67897.2 ± 2.6	13+,12+
14-3-3_2/Pin1/**MG1**$_2$	66007.4*	66008.1 ± 0.3	N/A
14-3-3_2/Pin1_2/**MG1**$_2$	68238.5*	68239.3 ± 0.7	14+,12+

7. NMR data

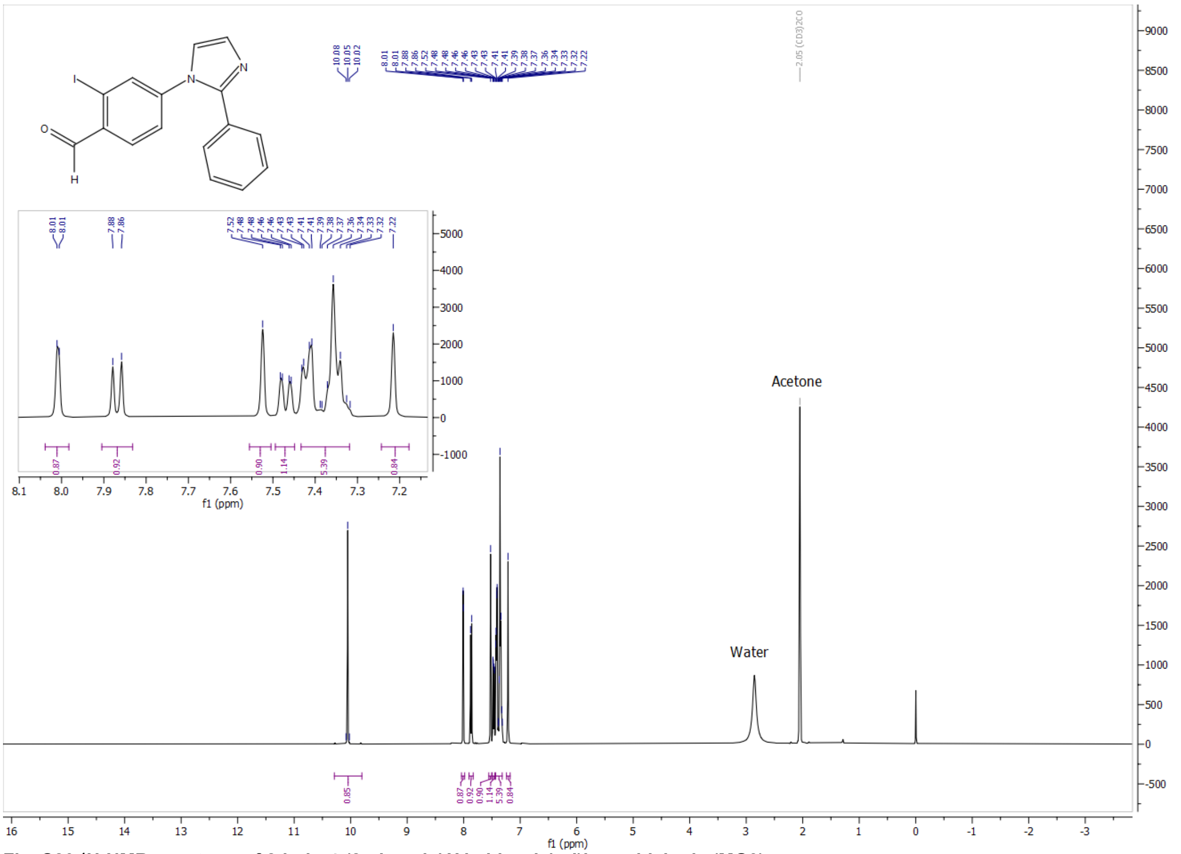

Fig. S20 ^{1}H-NMR spectrum of 2-iodo-4-(2-phenyl-1*H*-imidazol-1-yl)benzaldehyde (MG3).

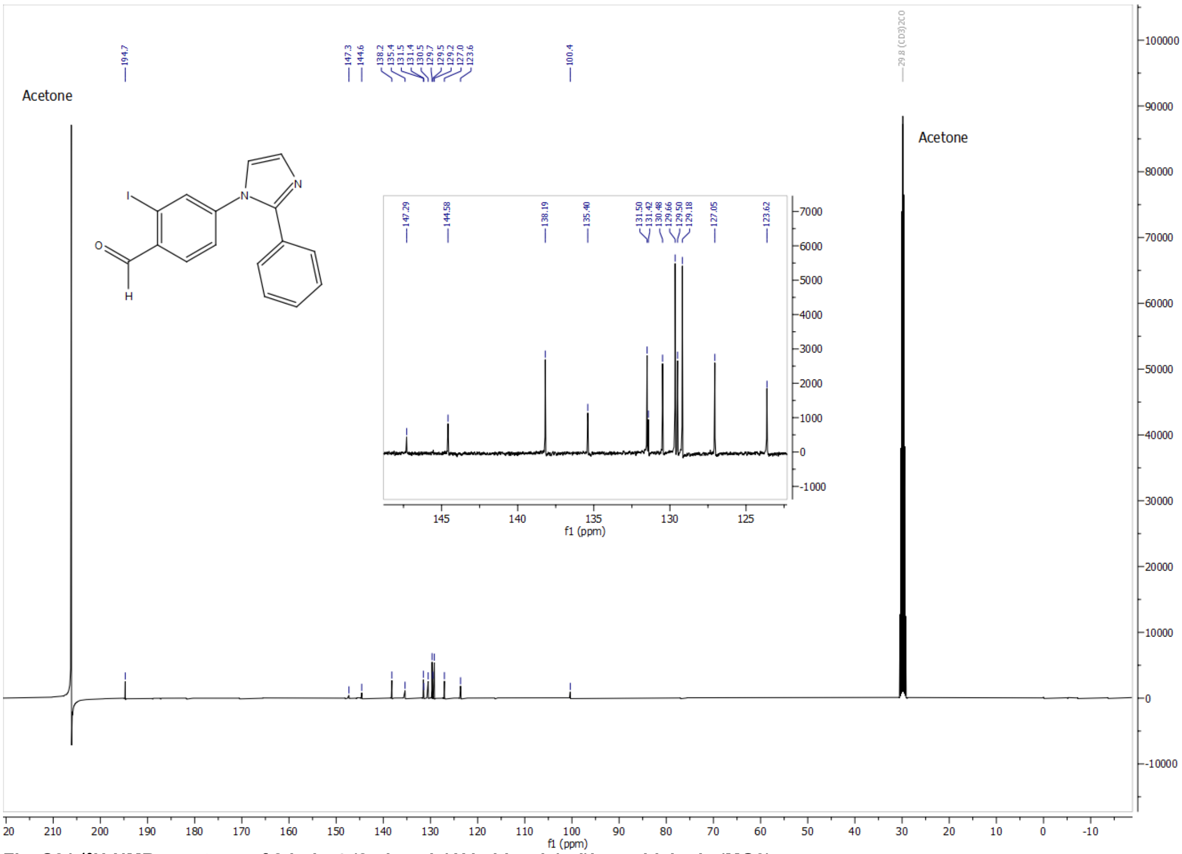

Fig. S21 ^{13}H-NMR spectrum of 2-iodo-4-(2-phenyl-1*H*-imidazol-1-yl)benzaldehyde (MG3).

8. LC-MS data

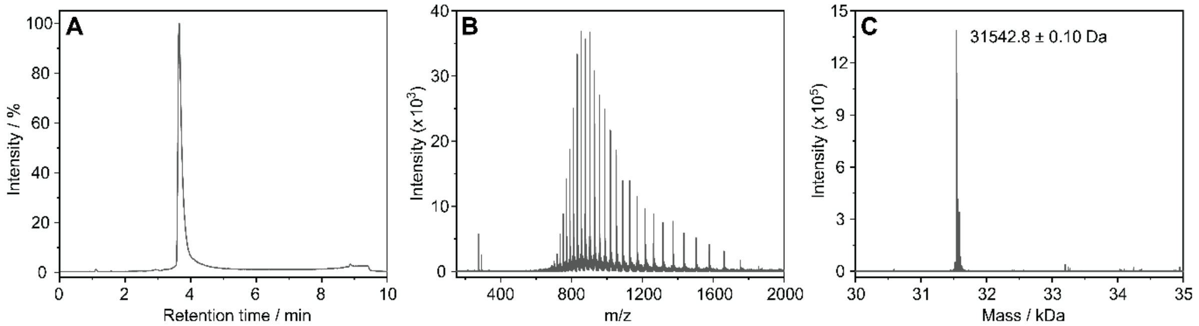

Fig. S22 Q-TOF LC-MS analysis of the full length 14-3-3γ protein. Chromatogram (A), mass spectrum (B), and deconvoluted mass spectrum (C).

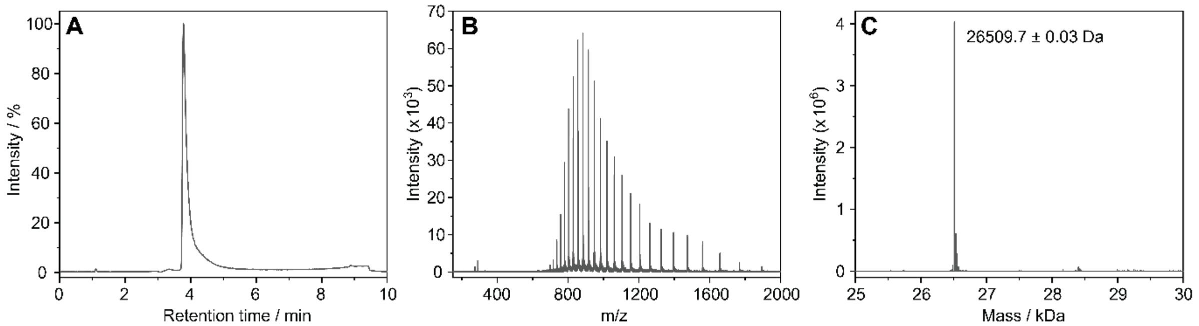

Fig. S23 Q-TOF LC-MS analysis of the 14-3-3σΔC protein. Chromatogram (A), mass spectrum (B), and deconvoluted mass spectrum (C).

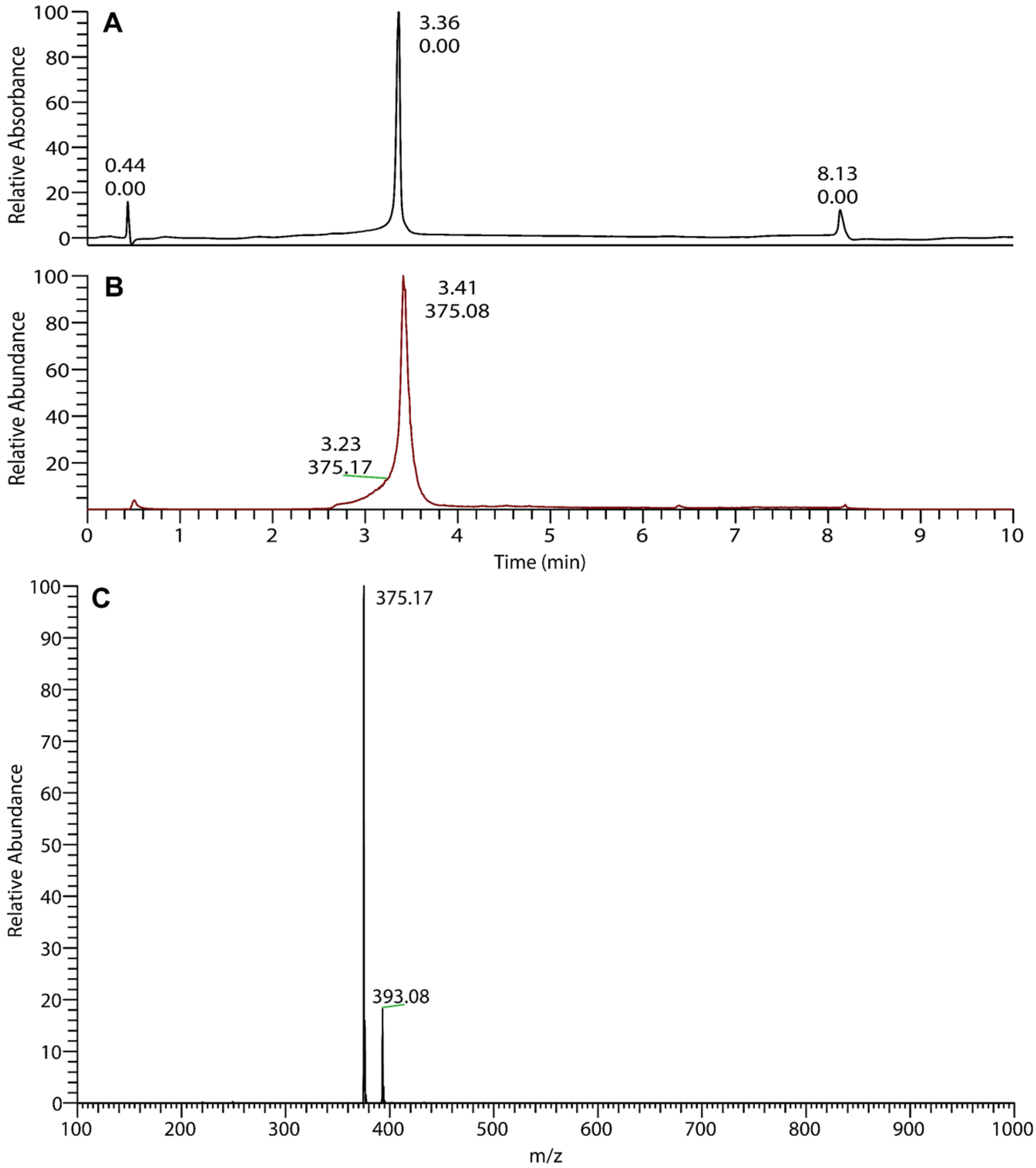

Fig. S24 LR LC-MS analysis of 2-iodo-4-(2-phenyl-1*H*-imidazol-1-yl)benzaldehyde (MG3). Chromatograms (absorbance (A), total ion count (B)), and mass spectrum (C).

9. References

1 P. J. Cossar, M. Wolter, L. Van Dijck, D. Valenti, L. M. Levy, C. Ottmann and L. Brunsveld, *J. Am. Chem. Soc.*, 2021, **143**, 8454–8464.

2 F. R. Leroux, B. Manteau, J. P. Vors and S. Pazenok, *Beilstein J. Org. Chem.*, DOI:10.3762/bjoc.4.13.

3 M. Wolter, D. Valenti, P. J. Cossar, S. Hristeva, L. M. Levy, T. Genski, T. Hoffmann, L. Brunsveld, D. Tzalis and C. Ottmann, *J. Med. Chem.*, 2021, **64**, 8423–8436.

4 M. R. Wilkins, E. Gasteiger, A. Bairoch, J. C. Sanchez, K. L. Williams, R. D. Appel and D. F. Hochstrasser, *Methods Mol. Biol.*, 1999, **112**, 531–552.

5 S. F. Altschul, T. L. Madden, A. A. Schäffer, J. Zhang, Z. Zhang, W. Miller and D. J. Lipman, *Nucleic Acids Res.*, 1997, **25**, 3389–3402.

6 C. Vonrhein, C. Flensburg, P. Keller, A. Sharff, O. Smart, W. Paciorek, T. Womack and G. Bricogne, *Acta Crystallogr. Sect. D Biol. Crystallogr.*, 2011, **67**, 293–302.

7 L. Potterton, J. Agirre, C. Ballard, K. Cowtan, E. Dodson, P. R. Evans, H. T. Jenkins, R. Keegan, E. Krissinel, K. Stevenson, A. Lebedev, S. J. McNicholas, R. A. Nicholls, M. Noble, N. S. Pannu, C. Roth, G. Sheldrick, P. Skubak, J. Turkenburg, V. Uski, F. Von Delft, D. Waterman, K. Wilson, M. Winn and M. Wojdyr, *Acta Crystallogr. Sect. D Struct. Biol.*, 2018, **74**, 68–84.

8 P. R. Evans and G. N. Murshudov, *Acta Crystallogr. Sect. D Biol. Crystallogr.*, 2013, **69**, 1204–1214.

9 P. R. Evans, *Acta Crystallogr. Sect. D Biol. Crystallogr.*, 2011, **67**, 282–292.

10 A. A. Lebedev, A. A. Vagin and G. N. Murshudov, *Acta Crystallogr. Sect. D Biol. Crystallogr.*, 2007, **64**, 33–39.

11 A. Vagin and A. Teplyakov, *Acta Crystallogr. Sect. D Biol. Crystallogr.*, 2010, **66**, 22–25.

12 F. Long, R. A. Nicholls, P. Emsley, S. Gražulis, A. Merkys, A. Vaitkus and G. N. Murshudov, *Acta Crystallogr. Sect. D Struct. Biol.*, 2017, **73**, 112–122.

13 P. Emsley, B. Lohkamp, W. G. Scott and K. Cowtan, *Acta Crystallogr. Sect. D Biol. Crystallogr.*, 2010, **66**, 486–501.

14 G. N. Murshudov, P. Skubák, A. A. Lebedev, N. S. Pannu, R. A. Steiner, R. A. Nicholls, M. D. Winn, F. Long and A. A. Vagin, *Acta Crystallogr. Sect. D Biol. Crystallogr.*, 2011, **67**, 355–367.

15 O. Kovalevskiy, R. A. Nicholls, F. Long, A. Carlon and G. N. Murshudov, *Acta Crystallogr. Sect. D Struct. Biol.*, 2018, **74**, 215–227.

16 P. V. Afonine, R. W. Grosse-Kunstleve, N. Echols, J. J. Headd, N. W. Moriarty, M. Mustyakimov, T. C. Terwilliger, A. Urzhumtsev, P. H. Zwart and P. D. Adams, *Acta Crystallogr. Sect. D Biol. Crystallogr.*, 2012, **68**, 352–367.

17 D. Liebschner, P. V. Afonine, M. L. Baker, G. Bunkoczi, V. B. Chen, T. I. Croll, B. Hintze, L. W. Hung, S. Jain, A. J. McCoy, N. W. Moriarty, R. D. Oeffner, B. K. Poon, M. G. Prisant, R. J. Read, J. S. Richardson, D. C. Richardson, M. D. Sammito, O. V. Sobolev, D. H. Stockwell, T. C. Terwilliger, A. G. Urzhumtsev, L. L. Videau, C. J. Williams and P. D. Adams, *Acta Crystallogr. Sect. D Struct. Biol.*, 2019, **75**, 861–877.

18 A. Klapars and S. L. Buchwald, *J. Am. Chem. Soc.*, 2002, **124**, 14844–14845.
